# Anthropogenic modification of forests means only 40% of remaining forests have high ecosystem integrity

**DOI:** 10.1038/s41467-020-19493-3

**Published:** 2020-12-08

**Authors:** H. S. Grantham, A. Duncan, T. D. Evans, K. R. Jones, H. L. Beyer, R. Schuster, J. Walston, J. C. Ray, J. G. Robinson, M. Callow, T. Clements, H. M. Costa, A. DeGemmis, P. R. Elsen, J. Ervin, P. Franco, E. Goldman, S. Goetz, A. Hansen, E. Hofsvang, P. Jantz, S. Jupiter, A. Kang, P. Langhammer, W. F. Laurance, S. Lieberman, M. Linkie, Y. Malhi, S. Maxwell, M. Mendez, R. Mittermeier, N. J. Murray, H. Possingham, J. Radachowsky, S. Saatchi, C. Samper, J. Silverman, A. Shapiro, B. Strassburg, T. Stevens, E. Stokes, R. Taylor, T. Tear, R. Tizard, O. Venter, P. Visconti, S. Wang, J. E. M. Watson

**Affiliations:** 1 Wildlife Conservation Society, Global Conservation Program, Bronx, New York, 10460 USA; 2 School of Earth and Environmental Sciences, University of Queensland, Brisbane, Australia; 3 Department of Biology, Carleton University, 1125 Colonel By Drive, Ottawa, ON K1S 5B6 Canada; 4 Wildlife Conservation Society Canada, 344 Bloor St W #204, Toronto, ON M5S 3A7 Canada; 5 United Nations Development Programme, One United Nations Plaza, New York, NY 10017 USA; 6 World Resources Institute, Washington, DC USA; 7 Global Earth Observation & Dynamics of Ecosystems Lab, School of Informatics, Computing, and Cyber Systems, Northern Arizona University, Flagstaff, AZ 86011 USA; 8 Landscape Biodiversity Lab, Ecology Department, Montana State University, Bozeman, MT 59717 USA; 9 Rainforest Foundation Norway, Mariboes gate 8, 0183 Oslo, Norway; 10 Global Wildlife Conservation, P.O. Box 129, Austin, TX 78767 USA; 11 School of Life Sciences, Arizona State University, P.O. Box 874501, Tempe, AZ 85287 USA; 12 Centre for Tropical Environmental and Sustainability Science, College of Science and Engineering, James Cook University, Cairns, QLD 4878 Australia; 13 Environmental Change Institute, School of Geography and the Environment, University of Oxford, Oxford, UK; 14 College of Science and Engineering, James Cook University, Townsville, Queensland, Australia; 15 School of Biological Sciences, University of Queensland, St. Lucia, QLD Australia; 16 The Nature Conservancy, Arlington, VA USA; 17 Jet Propulsion Laboratory, California Institute of Technology, Pasadena, CA 91109 USA; 18 World Wide Fund for Nature Germany, Space+Science, Berlin, Germany; 19 International Institute of Sustainability, Rio de Janeiro, 22460-320 Brazil; 20 Natural Resource and Environmental Studies Institute, University of Northern British Columbia, Prince George, Canada; 21 International Institute for Applied Systems Analysis, Laxenburg, Austria

**Keywords:** Conservation biology, Ecological modelling, Forest ecology

## Abstract

Many global environmental agendas, including halting biodiversity loss, reversing land degradation, and limiting climate change, depend upon retaining forests with high ecological integrity, yet the scale and degree of forest modification remain poorly quantified and mapped. By integrating data on observed and inferred human pressures and an index of lost connectivity, we generate a globally consistent, continuous index of forest condition as determined by the degree of anthropogenic modification. Globally, only 17.4 million km^2^ of forest (40.5%) has high landscape-level integrity (mostly found in Canada, Russia, the Amazon, Central Africa, and New Guinea) and only 27% of this area is found in nationally designated protected areas. Of the forest inside protected areas, only 56% has high landscape-level integrity. Ambitious policies that prioritize the retention of forest integrity, especially in the most intact areas, are now urgently needed alongside current efforts aimed at halting deforestation and restoring the integrity of forests globally.

## Abstract (web summary)

Mapping and quantifying degree of forest modification is critical to conserve and manage forests. Here the authors propose a new quantitative metric for landscape integrity and apply it to a global forest map, showing that less than half of the world’s forest cover has high integrity, most of which is outside nationally designed protected areas.

## Introduction

Deforestation is a major environmental issue^1^, but far less attention has been given to the degree of anthropogenic modification of remaining forests, which reduces ecosystem integrity and diminishes many of the benefits that these forests provide^2,3^. This is worrying since modification is potentially as significant as outright forest loss in determining overall environmental outcomes^4^. There is increasing recognition of this issue, for forests and other ecosystems, in synthesis reports by global science bodies such as the global assessment undertaken by the Intergovernmental Science-Policy Platform on Biodiversity and Ecosystem Services^5^, and it is now essential that the scientific community develop improved tools and data to facilitate the consideration of levels of integrity in decision-making. Mapping and monitoring this globally will provide essential information for coordinated global, national, and local policy-making, planning, and action, to help nations and other stakeholders achieve the Sustainable Development Goals (SDGs) and implement other shared commitments such as the United Nations Convention on Biological Diversity (CBD), Convention to Combat Desertification (UNCCD), and Framework Convention on Climate Change (UNFCCC).

Ecosystem integrity is foundational to all three of the Rio Conventions (UNFCCC, UNCCD, CBD)^6^. As defined by Parrish et al*.*^7^, it is essentially the degree to which a system is free from anthropogenic modification of its structure, composition, and function. Such modification causes the reduction of many ecosystem benefits, and is often also a precursor to outright deforestation^8,9^. Forests largely free of significant modification (i.e., forests having high ecosystem integrity), typically provide higher levels of many forest benefits than modified forests of the same type^10^, including; carbon sequestration and storage^11^, healthy watersheds^12^, traditional forest use^13^, contribution to local and regional climate processes^14^, and forest-dependent biodiversity^15–18^. Industrial-scale logging, fragmentation by infrastructure, farming (including cropping and ranching) and urbanization, as well as less visible forms of modification such as over-hunting, wood fuel extraction, and changed fire or hydrological regimes^19,20^, all degrade the degree to which forests still support these benefits, as well as their long-term resilience to climate change^10^. There can be trade-offs, however, between the benefits best provided by less-modified forests (e.g., regulatory functions such as carbon sequestration) and those production services that require some modification (e.g., timber production). These trade-offs can, at times, result in disagreement among stakeholders as to which forest benefits should be prioritized^21^.

In recent years, easily accessible satellite imagery and new analytical approaches have improved our ability to map and monitor forest extent globally^22–24^. However, while progress has been made in developing tools for assessment of global forest losses and gains, consistent monitoring of the degree of forest modification has proved elusive^25,26^.

Technical challenges include the detection of low intensity and unevenly distributed forest modification, the wide diversity of changes that comprise forest modification, and the fact that many changes are concealed by the forest canopy^25^. New approaches are emerging on relevant forest indicators, such as canopy height, canopy cover and fragmentation, and maps of different human pressures, which are used as proxies for impacts on forests^27–30^. Some binary measures of forest modification, such as Intact Forest Landscapes^31^ and wilderness areas^32^, have also been mapped at the global scale and used to inform policy, but do not resolve the degree of modification within remaining forests, which we aimed to do with this assessment.

**Fig. 1:**
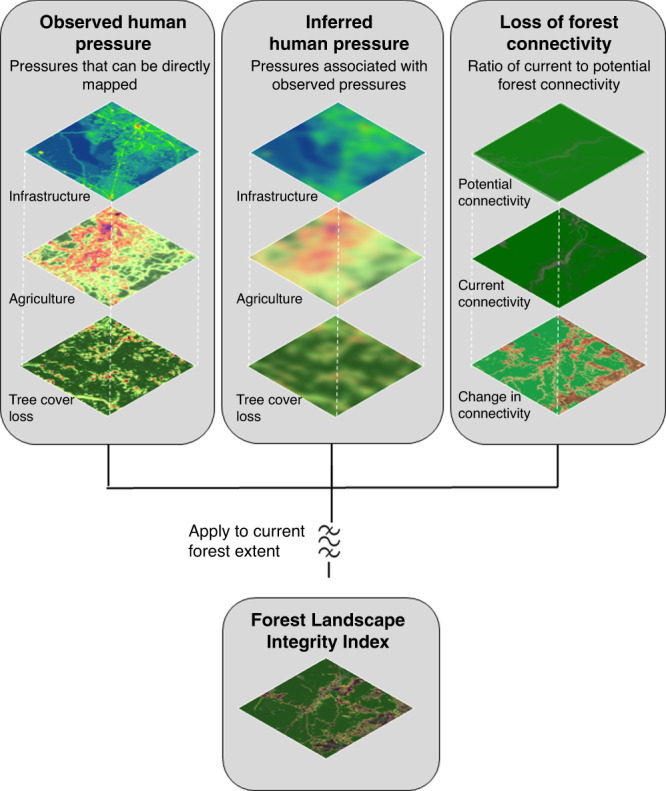
Methods used to construct the Forest Landscape Integrity Index. The Forest Landscape Integrity Index was constructed based on three main data inputs: (1) observed pressures (infrastructure, agriculture, tree cover loss), (2) inferred pressure modeled based on proximity to the observed pressures, and (3) change in forest connectivity.

Human activities influence the integrity of forests at multiple spatial scales, including intense, localized modifications such as road-building and canopy loss, more diffuse forms of change that are often spatially associated with these localized pressures (e.g., increased accessibility for hunting, other exploitation, and selective logging), and changes in spatial configuration that alter landscape-level connectivity. Previous studies have quantified several of these aspects individually^27–29^, but there is a need to integrate them to measure and map the overall degree of modification considering these landscape-level anthropogenic influences at each site. Here, we integrate data on forest extent defined as all woody vegetation taller than 5 m, following^23^, observed human pressures (e.g., infrastructure) which can be directly mapped using current datasets, other inferred human pressures (e.g., collection of forest materials) that occur in association with those that are observed but cannot be mapped directly, and alterations in forest connectivity, to create the Forest Landscape Integrity Index (FLII), that describes the degree of forest modification for the beginning of 2019 (Fig. 1). The result is a globally applicable, continuous-measure map of landscape-level forest integrity (hereafter, integrity), which offers a timely indicator of the status and management needs of Earth’s remaining forests. The results show there has been a huge loss of forest integrity. To give a global overview we summarize the results according to three simple, illustrative categories of integrity (which we term “high”, “medium”, and “low”) while noting that the underlying continuous index enables much finer distinctions to be made for detailed analysis in diverse contexts. This reveals around 40% of remaining forests have high forest integrity. Further, our methodological framework (Fig. 1) can be adapted to match local conditions at national or subnational scales and for different weightings to be applied.

## Results

**Fig. 2:**
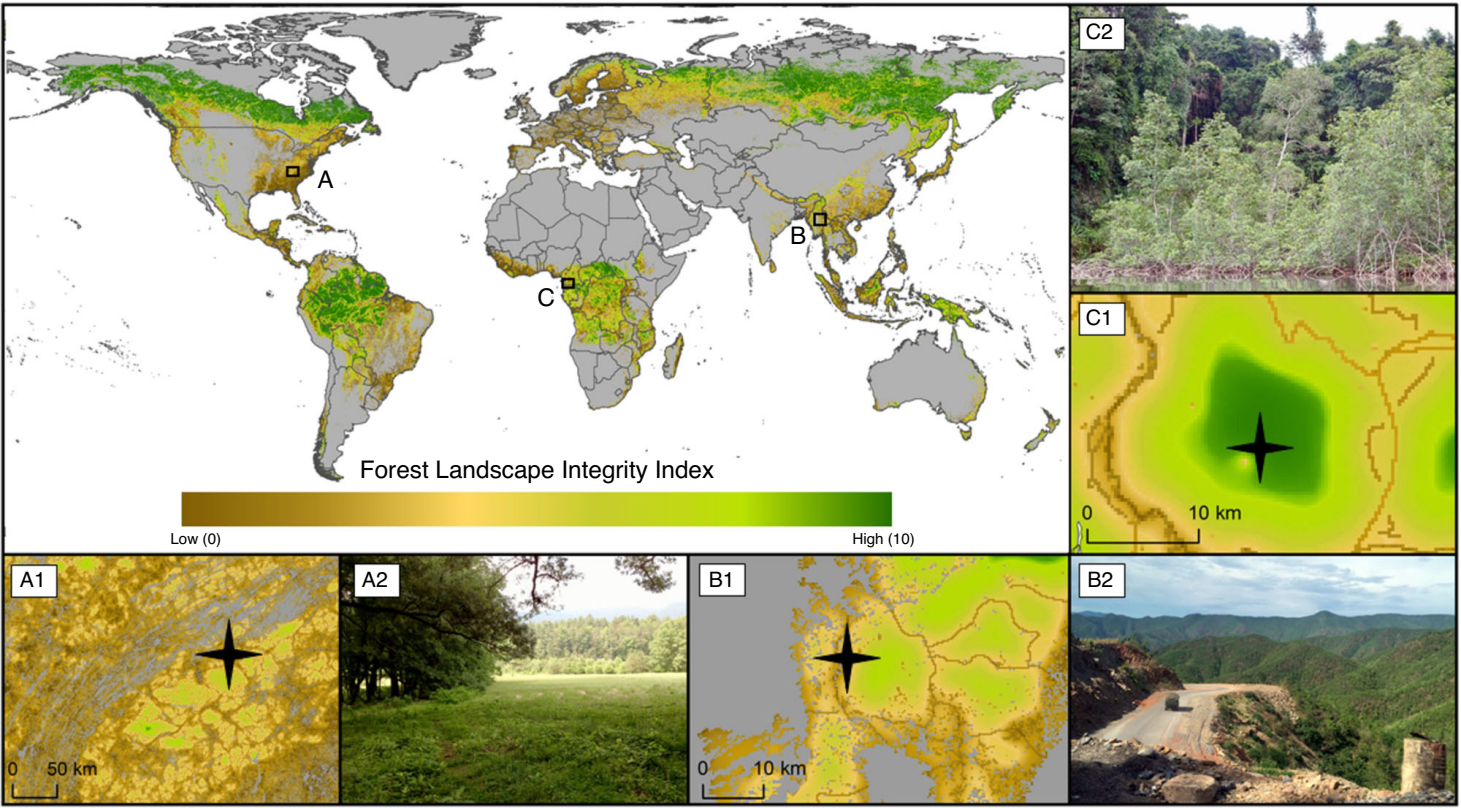
Forest Landscape Integrity Index map. A global map of Forest Landscape Integrity for the start of 2019. Three regions are highlighted including (**a**) Smoky Mountains National Park in Tennessee USA, (**b**) a region in Shan State Myanmar, and (**c**) Reserva Natural del Estuario del Muni in Equatorial Guinea. Maps A1–C1 shows the Forest Landscape Integrity Index for these locations. A2, B2, and C2 are photographs from within these regions: (A2) the edge of Smoky Mountains National Park; (B2) shows a logging truck passing through some partially degraded forest along a newly constructed highway in Shan Stat; and, (C3) shows an intact mangrove forest within Reserva Natural del Estuario del Muni, near the border with Gabon. The stars in (**a**), (**b**), and (**c**) indicate approximate location of where these photos were taken. All photos were taken by H.S.G.

Forest modification caused by human activity is both highly pervasive and highly variable across the globe (Fig. 2). We found 31.2% of forests worldwide are experiencing some form of observed human pressure, which included infrastructure, agriculture, and recent deforestation. Our models also inferred the likely occurrence of other pressures, and the impacts of lost connectivity, in almost every forest location (91.2% of forests), albeit sometimes at very low levels. Diverse, recognizable patterns of forest integrity can be observed in our maps at a range of scales, depending on the principal forms and general intensity of human activity in an area. Broad regional trends can be readily observed, for example, the overall gradient of decreasing human impact moving northwards through eastern North America (Fig. 2), and finer patterns of impact are also clearly evident, down to the scale of individual protected areas, forest concessions, settlements, and roads (Supplementary Fig. 2).

**Fig. 3:**
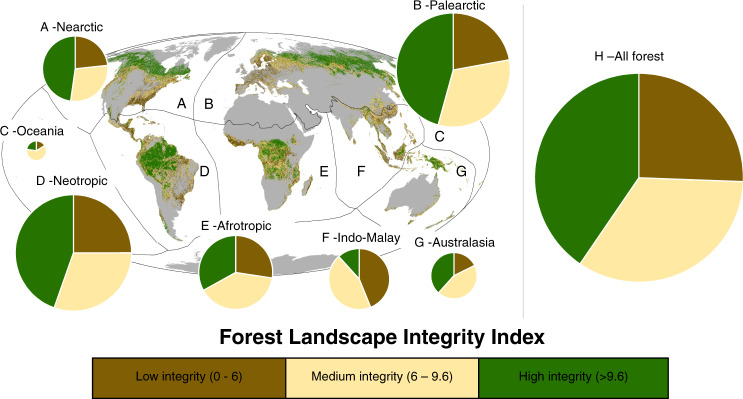
Forest Landscape Integrity Index map categorized into three illustrative classes. The Forest Landscape Integrity Index for 2019 categorized into three broad, illustrative classes and mapped across each biogeographic realm (**a**–**g**). The size of the pie charts indicates the relative size of the forests within each realm (**a**–**g**), and **h** shows all the world’s forest combined.

FLII scores range from 0 (lowest integrity) to 10 (highest). We discretized this range to define three broad illustrative categories: low (≤6.0); medium (>6.0 and <9.6); and high integrity (≥9.6) by benchmarking against reference locations worldwide (see Methods, Supplementary Table 4). Only 40.5% (17.4 million km^2^) of the forest was classified as having high integrity (Fig. 3; Table 1). Moreover, even in this category of high integrity 36% still showed at least a small degree of human modification. The remaining 59% (25.6 million km^2^) of the forest was classified as having low or medium integrity, including 25.6% (11 million km^2^) with low integrity (Fig. 3; Table 1). When we analyzed across biogeographical realms defined by^33^ not a single biogeographical realm of the world had more than half of its forests in the high category (Fig. 3; Table 1).

**Table 1:** Brief title: Forest Landscape Integrity Index scores for each biogeographic realm.

Biogeographic realm	Historical forest area	Current forest area	Proportion of forest remaining	FLII	High		Medium		Low	
					(9.6–10)		(6–9.6)		(0–6)	
	*km*^2^	*km*^2^	*%*	*Mean*	*km*^2^	*% of realm*	*km*^2^	*% of realm*	*km*^2^	*% of realm*
Afrotropic	9,071,897	7,362,740	81.2	7.34	2,450,953	33.3	2,903,483	39.4	2,008,304	27.3
Australasia	2,225,054	1,711,684	76.9	8.05	656,701	38.4	753,188	44	301,796	17.6
Indo-malayan	4,797,518	3,596,249	75.0	5.9	420,977	11.7	1,599,049	44.5	1,576,223	43.8
Neotropic	14,965,342	10,271,519	68.6	7.81	4,579,406	44.6	3,122,706	30.4	2,569,407	25
Oceania	30,746	23,389	76.1	7.66	5,279	22.6	14,331	61.3	3,780	16.2
Palearctic	16,524,088	12,172,668	73.7	8	5,571,997	45.8	3,910,629	32.1	2,690,042	22.1
Nearctic	9,756,589	7,794,117	79.9	7.84	3,716,855	47.7	2,257,518	29	1,819,744	23.3
Total	57,371,234	42,932,367	74.8	7.76	17,402,170		14,560,903		10,969,294	

A summary of the Forest Landscape Integrity Index scores for each biogeographic realm globally, measuring the mean score, in addition to the area and proportion of realm for each category of integrity. Scores are divided into three categories of integrity: high, medium, and low.

The biogeographical realms with the largest area of forest with high integrity are the Paleartic, particularly northern Russia, and the Neartic, in northern Canada, Rocky Mountains, and Alaska (Fig. 3). There are also large areas of forest with high integrity in the Neotropics, concentrated in the Amazon region, including within the Guianas, Atlantic forest in Brazil, southern Chile, and parts of Mesoamerica (Fig. 3, Table 1). The Afrotropic realm has significant areas with high integrity, particularly within the humid forests of central Africa (e.g., in Republic of Congo and Gabon) and in some of the surrounding drier forest/woodland belts (e.g., in South Sudan, Angola, and Mozambique) (Fig. 3). Some smaller patches occur in West Africa and Madagascar. In tropical Asia-Pacific, the largest tracts of forest with high integrity are in New Guinea. Smaller but still very significant tracts of forest with high integrity are also scattered elsewhere in each of the main forested regions, including parts of Sumatra, Borneo, Myanmar, and other parts of the Greater Mekong subregion.

Concentrations of the forest with low integrity are found in many regions including west and central Europe, the south-eastern USA, island and mainland South-East Asia west of New Guinea, the Andes, much of China and India, the Albertine Rift, West Africa, Mesoamerica, and the Atlantic Forests of Brazil (Fig. 3). The overall extent of forests with low integrity is greatest in the Paleartic realm, followed by the Neotropics, which are also those biogeographic realms with the largest forest cover (Table 1). The Indo-Malayan realm has the highest percentage with low integrity, followed by the Afrotropics (Fig. 3; Table 1).

**Fig. 4:**
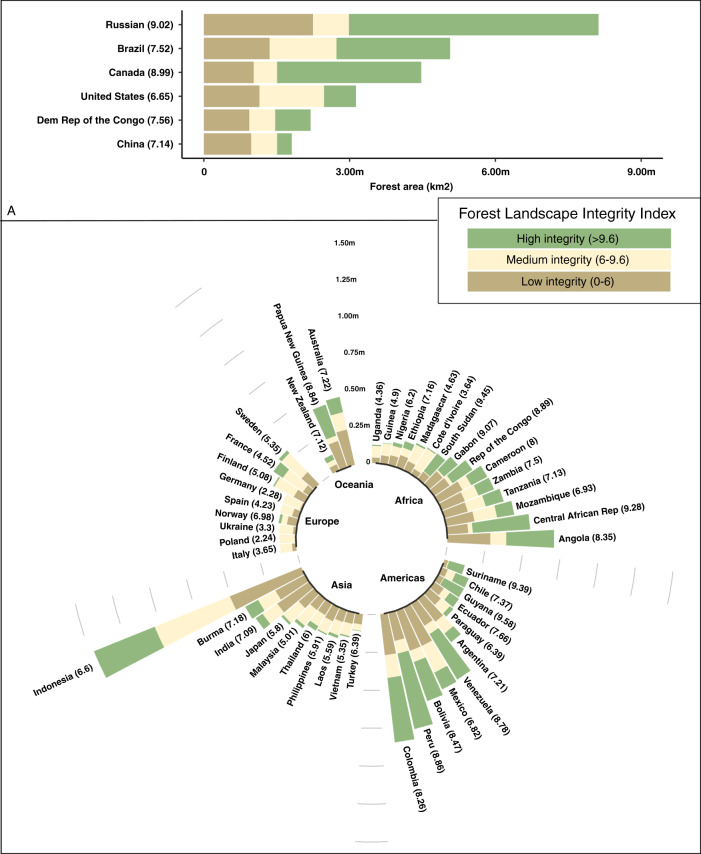
Forest Landscape Integrity Index map categorized into three illustrative classes for each major forested country. The Forest Landscape Integrity Index for 2019 categorized into three broad, illustrative classes for each major forested country in the world. (**a**) countries with a forest extent larger than 1 million km^2^, and (**b**) countries with forest extent between 1 million km^2^ and 100,000 km^2^ of forest. The size of the bar represents the area of a country’s forests.

These patterns result in variation of forest integrity scores in ways that allow objective comparisons to be made between locations and at a resolution relevant for policy and management planning, such as at national and sub-national scales. The global average FLII score is 7.76 (Table 1), representing a medium level of integrity. However, the average score across countries, disregarding their size, is 5.48, suggesting that low scores dominate in many of the smaller countries, and indeed a quarter of forested countries have a national average score < 4. National mean scores vary widely, ranging from >9 in Guyana, French Guiana, Gabon, Sudan, and South Sudan to <3 in Sierra Leone and many west European countries (see Fig. 4. and Supplementary Table 5 for a full list of countries). Provinces and other sub-national units vary even more widely (see Supplementary Fig. 2 and Supplementary Table 6)

Over one-quarter (26.1%) of all forests with high integrity fall within protected areas, compared to just 13.1% of low and 18.5% of medium integrity forests respectively. For all forests that are found within nationally designated protected areas (around 20% of all forests globally), we found the proportions of low, medium, and high integrity forests were 16.8%, 30.3%, and 52.8%, respectively (Table 2). Within the different protected area categories, we typically found that there was more area within the high integrity category versus the medium and low except for Category V (protected landscape/seascape) (Table 2). However, with 47.1% of forests within protected areas having low to medium integrity overall, it is clear that forests considered protected are already often fairly modified (Table 2). Even though they are quite modified, some of these forests might still have high conservation importance, such as containing endangered species.

**Table 2:** Brief title: Forest Landscape Integrity Index scores for different types of protected areas.

Protected area category	Total forest	FLII	High (score 9.6–10)		Medium (score 6–9.6)		Low (score 0–6)	
	*km*^2^	*Mean*	*km*^2^	*% of protected area*	*km*^2^	*% of protected area*	*km*^2^	*% of protected area*
Ia (strict nature reserve)	439,082	9.27	304,329	69.31	106,703	24.3	28,049	6.39
Ib (wilderness area)	367,330	9.22	240,453	65.46	102,096	27.79	24,780	6.75
II (national park)	1,900	9.14	1,223,138	64.38	540,805	28.46	136,056	7.16
III (natural monument or feature)	113,805	8.49	54,476	47.87	40,021	35.17	19,308	16.97
IV (habitat/species management area)	838,707	8.69	432,828	51.61	268,027	31.96	137,850	16.44
V (protected landscape/seascape)	840,919	6.4	224,491	26.7	295,769	35.17	320,658	38.13
VI (Protected area with sustainable use of natural resources)	1,472,278	9.21	1,026,169	69.7	344,617	23.41	101,491	6.89
Not Applicable / Not Assigned / Not Reported	2,613,541	8.29	1,030,430	39.42	906,745	34.69	676,365	25.88
All Protected Areas	8,585,661	8.55	4,536,314	52.83	2,694,784	30.34	1,444,562	16.82

A summary of the Forest Landscape Integrity Index scores for each type of protected area designation based on the IUCN Protected Areas categories measuring mean score, in addition to the area and proportion of realm for each category of integrity. Scores are divided into three categories of integrity: high, medium, and low.

## Discussion

By providing a transparent and defensible methodological framework, and by taking advantage of global data on forest extent, human drivers of forest modification, and changes in forest connectivity, our analysis paints a sobering picture of the extent of human impacts on the world’s forests. This analysis enables the changes that degrade many forest values to be visualized in a way for policymakers and decision-makers to see where forests that survive in good condition are found. By integrating data on multiple human pressures that are known to modify forests, our analysis moves global quantification beyond the use of simple categories, or solely using pressure indicators as proxies for integrity, to a more nuanced depiction of this issue as a continuum, recognizing that not all existing forests are in the same condition. Our analysis reveals that severe and extensive forest modification has occurred across all biogeographic regions of the world. Consequently, indices only using forest extent may inadequately capture the true impact of human activities on forests, and are insensitive to many drivers of forest modification and the resulting losses of forest benefits.

A plan is clearly needed to put in place retention strategies for the remaining forests with high integrity, tailored towards the context in each country or jurisdiction and its different forest types^34–36^, because such areas are known to hold exceptional value. Avoiding the loss of integrity is a better strategy than aiming to restore forest condition after it is lost, because restoration is more costly, has a risk of failure, and is unlikely to lead to full recovery of benefits^5^. For the forests with the highest integrity to be retained they should ideally be mapped using nationally appropriate criteria by the countries that hold them, formally recognized, prioritized in spatial plans, and placed under effective management (e.g., protected areas and other effective conservation areas, lands under Indigenous control, etc.). These forests must be protected from industrial development impacts that degrade them through sensible public and private sector policy that is effective at relevant scales^13,37^. Our global assessment reveals where these places are found, and can be refined at more local scales where better data are available.

Around a third of global forests had already been cleared by 2000^38^, and we show that at least 59% of what remains has low or medium integrity, with > 50% falling in these two broad categories in every biogeographical realm. These levels of human modification result partly from the large areas affected by relatively diffuse anthropogenic pressures whose presence is inferred near forest edges, and by lost connectivity. We also map a surprising level of more localized, observed pressures, such as infrastructure and recent forest loss, which are seen in nearly a third of forested pixels worldwide.

Conservation strategies in these more heavily human-modified forests should focus on securing any remaining fragments of forests in good condition, proactively protecting those forests most vulnerable to further modification^8^ and planning where restoration efforts might be most effective^39–41^. In addition, effective management of production forests is needed to sustain yields without further worsening their ecological integrity^42^. More research is required on how to prioritize, manage, and restore forests with low to medium integrity^41,43^, and the FLII presented here might prove useful for this, for example, by helping prioritize where the best returns on investment are, in combination with other sources of data (e.g., carbon)^44^.

Loss of forest integrity severely compromises many benefits of forests that are central to achieving multiple Sustainable Development Goals and other societal targets^45,46^. Therefore, governments must adopt policies and strategies to retain and restore the ecological integrity of their forests, whilst ensuring that the solutions are also economically viable, socially equitable, and politically acceptable within complex and highly diverse local contexts. This is an enormous challenge and our efforts to map the degree of forest modification are designed both to raise awareness of the importance of the issue, and to support implementation through target setting, evidence-based planning, and enhanced monitoring efforts.

Whilst policy targets for halting deforestation are generally precise and ambitious, only vague targets are typically stipulated around reducing levels of forest modification^10,47^. We urgently need SMART (specific, measurable, achievable, realistic, and time-bound) goals, targets, and indicators for maintaining and restoring forest integrity that directly feeds into higher-level biodiversity, climate, land degradation, and sustainable development goals^48^. Forest specific targets could be included within an over-arching target on ecosystems within the post-2020 Global Biodiversity Framework, which is currently being negotiated among Parties to the CBD^49^. This target needs to be outcome-focused and address both the extent and the integrity of ecosystems (e.g., using FLII for forests), in a way that enables quantitative, measurable goals to be set and reported on, but allows flexibility for implementation between Parties. The index we provide here could be easily updated annually and utilized by nations as a way to report the state of their forests.

In addition to broader goals in global frameworks, the retention and restoration of forest integrity should also be addressed in nationally-defined goals embodied in, and aligned between, Nationally Determined Contributions under the UNFCCC, efforts to stop land degradation and achieve land degradation neutrality under the UNCCD, and National Biodiversity Strategy and Action Plans under the CBD. Since no single metric can capture all aspects of a country’s environmental values, efforts to conserve high levels of forest integrity should be complemented by consideration of areas that support important values according to other measures (e.g., Key Biodiversity Areas^50^ and notable socio-cultural landscapes).

A key management tool for maintaining and improving forest integrity is protected areas^10^. We found over a quarter of forests with high integrity are within protected areas, showing that this importance has been widely recognized by some national authorities. However, we also found that nearly half of the forests within protected areas have medium or low integrity. This result aligns with other studies such as Jones et al*.*^51^ that found a third of protected areas had high human pressure within them. Compared with more restricted protected areas (e.g., category I), there was a broad trend of decreasing forest integrity in protected area categories that allows more human use, with particularly low mean scores and high percentages of the forest with low integrity in Category V (Protected Landscapes/Seascapes). The exception is category VI, which includes indigenous and community protected areas, some of which contain very extensive areas with low human population pressure, and for which mean integrity scores are comparable to those in category I. Some of these differences probably represent differences at the time of establishment, so time series or quasi-experimental methods are needed to clarify the degree to which the various categories are effective in mitigating threats to integrity, as suggested by Fa et al*.*^52^.

The overall level and pervasiveness of impacts on Earth’s remaining forests is likely even more severe than our findings suggest, because some input data layers, despite being the most comprehensive available, are still incomplete as there are lags between increases in human pressures and our ability to capture them in spatial datasets e.g., infrastructure^53,54^, (see also Supplementary Note 5 and Supplementary Fig. 1). For example, roads and seismic lines used for natural resource exploration and extraction in northern boreal regions of Canada, are not fully reflected in global geospatial datasets (Supplementary Fig. 1; see also^55^) The over-exploitation of high socio-economic value animals and plants may be quite varied across nations and region, driven by complex social, cultural, economic and governance factors e.g.^56,57^, which are difficult to model spatially but as these data become available, they could be included in further updates of the index. Adding a temporal dimension of the index is an important next step, as it will be possible to start to assess the drivers and underlying caused leading to intact forest erosion which clearly requires further research attention. Furthermore, because natural fires are such an important part of the ecology of many forest systems (e.g., boreal forests) and it is not possible to consistently identify anthropogenic fires from natural fires at a global scales^58^ we have taken a strongly conservative approach to fire in our calculations, treating all tree cover loss in 10 km pixels where fire was the dominant driver as temporary, and not treating such canopy loss as evidence of observed human pressure. Varying these assumptions where human activity is shown to be causing permanent tree cover losses, increasing fire return frequencies, or causing fire in previously fire-free systems would result in lower forest extent and/or lower forest integrity scores in some regions than we report.

We map forest integrity based on quantifiable processes over the recent past (since 2000). In some areas modification that occurred prior to this (e.g., historical logging) is not detectable by our methods but may have influenced the present-day integrity of the forest so, in such cases, we may overestimate forest integrity. This is another reason why our index should be considered as conservative, and we, therefore, recommend that the index be used alongside other lines of evidence to determine the absolute level of the ecological integrity of a given area. Moreover, the definition of forest in this study is all woody vegetation taller than 5 m, following^23^ and hence includes not only naturally regenerated forests but also tree crops, planted forests, wooded agroforests, and urban tree cover in some cases. Users should be mindful of this when interpreting the results, especially when observing areas with low forest integrity scores. Inspection of the results for selected countries with reliable plantation maps^59^ shows that the great majority of planted forests have low forest integrity scores, because they are invariably associated with dense infrastructure, frequent canopy replacement, and patches of farmland.

We note our measure of forest integrity does not address past, current, and future climate change. As climate change affects forest conditions both directly and indirectly, this is a clear shortfall and needs research attention. The same is true for invasive species, as there are no globally coherent data on the ranges of those invasive species that degrade forest ecosystems, although this issue is indirectly addressed since the presence of many invasive species is likely spatially correlated with the human pressures that we use as drivers in our model^27^. We estimated the likely occurrence of damage caused by inferred pressures using a distance function; this function could be tailored to particular contexts, such as the presence of high-value species or unusually difficult terrain, if training data were available. As global data become available it would also be valuable to incorporate data on other drivers of forest integrity loss. Future research might enable the inclusion of governance effectiveness as a factor in our model, because there are potentially contexts (e.g., well-managed protected areas and community lands, production forests under “sustainable forest management”) where the impacts associated with the human pressures we base our map on are at least partially ameliorated^42^, and enhanced governance is also likely to be a significant component of some future strategies to maintain and enhance forest integrity.

The framework we present is now being tailored for use at smaller scales, ranging from regional to national and sub-national scales, and even to individual management units, through the development of a cloud-based online tool. Forest definitions and the relative weights of the global parameters we use can be adjusted to fit local contexts and, in many cases, better local data could be substituted, or additional variables incorporated. This would not only increase the precision of the index in representing local realities, but also the degree of ownership amongst national and local policymakers and stakeholders whose decisions are so important in determining forest management trajectories.

## Methods

To produce our global Forest Landscape Integrity Index (FLII), we combined four sets of spatially explicit datasets representing: (i) forest extent^23^; (ii) observed pressure from high impact, localized human activities for which spatial datasets exist, specifically: infrastructure, agriculture, and recent deforestation^27^; (iii) inferred pressure associated with edge effects^27^, and other diffuse processes, (e.g., activities such as hunting and selective logging)^27^ modeled using proximity to observed pressures; and iv) anthropogenic changes in forest connectivity due to forest loss^27^ (see Supplementary Table 1 for data sources). These datasets were combined to produce an index score for each forest pixel (300 m), with the highest scores reflecting the highest forest integrity (Fig. 1), and applied to forest extent for the start of 2019. We use globally consistent parameters for all elements (i.e., parameters do not vary geographically). All calculations were conducted in Google Earth Engine (GEE)^60^.

### Forest extent

We derived a global forest extent map for 2019 by subtracting from the Global Tree Cover product for 2000^23^ annual Tree Cover Loss 2001–2018, except for losses categorized by Curtis and colleagues^24^ as those likely to be temporary in nature (i.e., those due to fire, shifting cultivation and rotational forestry). We applied a canopy threshold of 20% based on related studies e.g.^31,61^, and resampled to 300 m resolution and used this resolution as the basis for the rest of the analysis (see Supplementary Note 1 for further methods).

### Observed human pressures

We quantify observed human pressures (P) within a pixel as the weighted sum of impact of infrastructure (I; representing the combined effect of 41 types of infrastructure weighted by their estimated general relative impact on forests (Supplementary Table 3), agriculture (A) weighted by crop intensity (indicated by irrigation levels), and recent deforestation over the past 18 years (H; excluding deforestation from fire, see Discussion). Specifically, for pixel i:

1 $${\mathrm{P}}_{\mathrm{i}} = {\mathrm{exp}}\left( { - {\upbeta}_1{\mathrm{I}}_{\mathrm{i}}} \right) + {\mathrm{exp}}\left( { - {\upbeta}_2{\mathrm{A}}_{\mathrm{i}}} \right) + {\mathrm{exp}}\left( { - {\upbeta}_3{\mathrm{H}}_{\mathrm{i}}} \right)$$

whereby the values of β were selected so that the median of the non-zero values for each component was 0.75. This use of exponents is a way of scaling variables with non-commensurate units so that they can be combined numerically, while also ensuring that the measure of observed pressure is sensitive to change (increase or decrease) in the magnitude of any of the three components, even at large values of I, A, or H. This is an adaptation of the Human Footprint methodology^62^. See Supplementary Note 3 for further details.

### Inferred human pressures

Inferred pressures are the diffuse effects of a set of processes for which directly observed datasets do not exist, that include microclimate and species interactions relating to the creation of forest edges^63^ and a variety of intermittent or transient anthropogenic pressures such as selective logging, fuelwood collection, hunting; spread of fires and invasive species, pollution, and livestock grazing^64–66^. We modeled the collective, cumulative impacts of these inferred effects through their spatial association with observed human pressure in nearby pixels, including a decline in effect intensity according to distance, and partitioning into stronger short-range and weaker long-range effects. The inferred pressure (P′) on pixel *i* from source pixel *j* is:

2 $$P\prime _{i,j} = P_j\left( {w_{i,j} + v_{i,j}} \right)$$

where w_*i,j*_ is the weighting given to the modification arising from short-range pressure, as a function of distance from the source pixel, and v_*i,j*_ is the weighting given to the modification arising from long-range pressures.

Short-range effects include most of the processes listed above, which together potentially affect most biophysical features of a forest, and predominate over shorter distances. In our model, they decline exponentially, approach zero at 3 km, and are truncated to zero at 5 km (see Supplementary Note 4).

3 $$\begin{array}{l}{\mathrm{w}}_{i,j} = \alpha \,{\mathrm{exp}}( - \lambda {\mathrm{d}}_{i,j})\,\,\,\,\,\,[{\mathrm{for}}\,{\mathrm{d}}_{{\mathrm{i,j}}} \le {\mathrm{5km}}]\\ {\mathrm{w}}_{i,j} = {\mathrm{0}}\,\,\,\,\,\,\,\,\,\,\,\,\,\,\,\,\,\,\,\,\,\,\,\,\,\,\,\,\,\,\,\,\,\,[{\mathrm{for}}\,{\mathrm{d}}_{i,j} > {\mathrm{5km}}]\end{array}$$

where α is a constant set to ensure that the sum of the weights across all pixels in the range is 1.85 (see below), λ is a decay constant set to a value of 1 (see^67^ and other references in Supplementary Note 4) and d_*i,j*_ is the Euclidean distance between the centers of pixels *i* and *j* expressed in units of km.

Long-range effects include over-exploitation of high socio-economic value animals and plants, changes to migration and ranging patterns, and scattered fire and pollution events. We modeled long-range effects at a uniform level at all distances below 6 km and they then decline linearly with distance, conservatively reaching zero at a radius of 12 km^65,68^ (and other references in Supplementary Note 4):

4 $$\begin{array}{l}{\mathrm{v}}_{i,j} = \gamma \,\,\,\,\,\,\,\,\,\,\,\,\,\,\,\,\,\,\,\,\,\,\,\,\,\,\,\,\,\,\,\,\,[for\,d_{i,j} \le 6km]\\ {\mathrm{v}}_{i,j} = \gamma \left( {12 - d_{i,j}} \right)/6\,\,\,\,[{\mathrm{for}}\,6{\mathrm{km}}\, < \,{\mathrm{d}}_{i,j} \le 12{\mathrm{km}}]\\ {\mathrm{v}}_{i,j} = 0\,\,\,\,\,\,\,\,\,\,\,\,\,\,\,\,\,\,\,\,\,\,\,\,\,\,\,\,\,\,\,\,[for\,{\mathrm{d}}_{i,j} > 12{\mathrm{km}}]\end{array}$$

where γ is a constant set to ensure that the sum of the weights across all pixels in the range is 0.15 and d_*i,j*_ is the Euclidean distance between the centers of pixels *i* and *j*, expressed in kilometers.

The form of the weighting functions for short- and long-range effects and the sum of the weights (α + γ) were specified based on a hypothetical reference scenario where a straight forest edge is adjacent to a large area with uniform human pressure, and ensuring that in this case total inferred pressure immediately inside the forest edge is equal to the pressure immediately outside, before declining with distance. γ is set to 0.15 to ensure that the long-range effects conservatively contribute no more than 5% to the final index in the same scenario, based on expert opinion and supported e.g., Berzaghi et al*.*^69^ regarding the approximate level of impact on values that would be affected by severe defaunation and other long-range effects.

The aggregate effect from inferred pressures (Q) on pixel *i* from all *n* pixels within range (*j* = *1* to *j* = *n*) is then the sum of these individual, normalized, distance-weighted pressures, i.e.,

5 $$Q_i = \mathop {\sum}_{j=1}^{n} {P{\prime}_{i,j}}$$

### Loss of forest connectivity

Average connectivity of forest around a pixel was quantified using a method adapted from Beyer et al.^70^. The connectivity C_*i*_ around pixel *i* surrounded by n other pixels within the maximum radius (numbered *j* = 1, 2…n) is given by:

6 $${\mathrm{C}}_i = \mathop {\sum}_{j=1}^{n} {\left( {{\mathrm{F}}_j{\mathrm{G}}_{i,j}} \right)}$$

where F_j_ is the forest extent is a binary variable indicating if forested (1) or not (0) and G_*i,j*_ is the weight assigned to the distance between pixels *i* and *j*. G_*i,j*_ uses a normalized Gaussian curve, with σ = 20 km and distribution truncated to zero at 4σ for computational convenience (see Supplementary Note 2). The large value of σ captures landscape connectivity patterns operating at a broader scale than processes captured by other data layers. C_*i*_ ranges from 0 to 1 (C_*i*_∈[0,1]).

Current Configuration (CC_*i*_) of forest extent in pixel i was calculated using the final forest extent map and compared to the Potential Configuration (PC) of forest extent without extensive human modification, so that areas with naturally low connectivity, e.g., coasts and natural vegetation mosaics, are not penalized. PC was calculated from a modified version of the map of Laestadius *et al*^38^. and resampled to 300 m resolution (see Supplementary Note 2 for details). Using these two measures, we calculated Lost Forest Configuration (LFC) for every pixel as:

7 $${\mathrm{LFC}}_i = 1 - \left( {{\mathrm{CC}}_i/{\mathrm{PC}}_i} \right)$$

Values of CC_*i*_/PC_*i*_ > 1 are assigned a value of 1 to ensure that LFC is not sensitive to apparent increases in forest connectivity due to inaccuracy in estimated potential forest extent – low values represent least loss, high values greatest loss (LFC_*i*_∈[0,1]).

### Calculating the Forest Landscape Integrity Index

The three constituent metrics, LFC, P, and Q, all represent increasingly modified conditions the larger their values become. To calculate a forest integrity index in which larger values represent less degraded conditions we, therefore, subtract the sum of those components from a fixed large value (here, 3). Three was selected as our assessment indicates that values of LFC + P + Q of 3 or more correspond to the most severely degraded areas. The metric is also rescaled to a convenient scale (0-10) by multiplying by an arbitrary constant (10/3). The FLII for forest pixel *i* is thus calculated as:

8 $${\mathrm{FLII}}_i = \left[ {10/3} \right] (3 - {\mathrm{min}}(3,\,[P_i + Q_i + {\mathrm{LFC}}_i]))$$

where FLII_*i*_ ranges from 0 to 10, forest areas with no modification detectable using our methods scoring 10 and those with the most scoring 0.

### Illustrative forest integrity classes

Whilst a key strength of the index is its continuous nature, the results can also be categorized for a range of purposes. In this paper three illustrative classes were defined, mapped, and summarized to give an overview of broad patterns of integrity in the world’s forests. The three categories were defined as follows.

High Forest Integrity (scores ≥ 9.6) Interiors and natural edges of more or less unmodified naturally regenerated (i.e., non-planted) forest ecosystems, comprised entirely or almost entirely of native species, occurring over large areas either as continuous blocks or natural mosaics with non-forest vegetation; typically little human use other than low-intensity recreation or spiritual uses and/or low-intensity extraction of plant and animal products and/or very sparse presence of infrastructure; key ecosystem functions such as carbon storage, biodiversity, and watershed protection and resilience expected to be very close to natural levels (excluding any effects from climate change) although some declines possible in the most sensitive elements (e.g., some high value hunted species).

Medium Forest Integrity (scores > 6.0 but <9.6) Interiors and natural edges of naturally regenerated forest ecosystems in blocks smaller than their natural extent but large enough to have some core areas free from strong anthropogenic edge effects (e.g., set-asides within forestry areas, fragmented protected areas), dominated by native species but substantially modified by humans through a diversity of processes that could include fragmentation, creation of edges and proximity to infrastructure, moderate or high levels of extraction of plant and animal products, significant timber removals, scattered stand-replacement events such as swidden and/or moderate changes to fire and hydrological regimes; key ecosystem functions such as carbon storage, biodiversity, watershed protection and resilience expected to be somewhat below natural levels (excluding any effects from climate change).

Low Forest Integrity (score ≤ 6.0): Diverse range of heavily modified and often internally fragmented ecosystems dominated by trees, including (i) naturally regenerated forests, either in the interior of blocks or at edges, that have experienced multiple strong human pressures, which may include frequent stand-replacing events, sufficient to greatly simplify the structure and species composition and possibly result in significant presence of non-native species, (ii) tree plantations and, (iii) agroforests; in all cases key ecosystem functions such as carbon storage, biodiversity, watershed protection and resilience expected to be well below natural levels (excluding any effects from climate change).

The numerical category boundaries were derived by inspecting FLII scores for a wide selection of benchmark locations whose forest integrity according to the category definitions was known to the authors, see text S6 and Table S4.

### Protected areas analysis

Data on protected area location, boundary, and year of the inscription were obtained from the February 2018 World Database on Protected Areas^71^. Following similar global studies e.g.^72^, we extracted protected areas from the WDPA database by selecting those areas that have a status of “designated”, “inscribed”, or “established”, and were not designated as UNESCO Man and Biosphere Reserves. We included only protected areas with detailed geographic information in the database, excluding those represented as a point only. To assess the integrity of the protected forest, we extracted all 300 m forest pixels that were at least 50% covered by a formally protected area and measured the average FLII score.

### Reporting summary

Further information on research design is available in the Nature Research Reporting Summary linked to this article.

## Supplementary information

Supplementary Information

Reporting Summary

## Data Availability

The authors declare that all data supporting the findings of this study are available at www.forestlandscapeintegrity.com. The datasets used to develop the Forest Landscape Integrity Index can be found at the following websites: tree cover and loss http://earthenginepartners.appspot.com/science-2013-global-forest, tree cover loss driver https://data.globalforestwatch.org/datasets/f2b7de1bdde04f7a9034ecb363d71f0e, potential forest cover https://data.globalforestwatch.org/datasets/potential-forest-coverage ESA-CCI Land Cover https://maps.elie.ucl.ac.be/CCI/viewer/index.php Open Street Maps https://www.openstreetmap.org, croplands https://lpdaac.usgs.gov/news/release-of-gfsad-30-meter-cropland-extent-products/, surface water https://global-surface-water.appspot.com/, protected areas https://www.protectedplanet.net/en.

## Code availability

The code for Google Earth Engine is available upon any reasonable request.
