# Author Correction: Anthropogenic modification of forests means only 40% of remaining forests have high ecosystem integrity

**DOI:** 10.1038/s41467-021-20999-7

**Published:** 2021-01-20

**Authors:** H. S. Grantham, A. Duncan, T. D. Evans, K. R. Jones, H. L. Beyer, R. Schuster, J. Walston, J. C. Ray, J. G. Robinson, M. Callow, T. Clements, H. M. Costa, A. DeGemmis, P. R. Elsen, J. Ervin, P. Franco, E. Goldman, S. Goetz, A. Hansen, E. Hofsvang, P. Jantz, S. Jupiter, A. Kang, P. Langhammer, W. F. Laurance, S. Lieberman, M. Linkie, Y. Malhi, S. Maxwell, M. Mendez, R. Mittermeier, N. J. Murray, H. Possingham, J. Radachowsky, S. Saatchi, C. Samper, J. Silverman, A. Shapiro, B. Strassburg, T. Stevens, E. Stokes, R. Taylor, T. Tear, R. Tizard, O. Venter, P. Visconti, S. Wang, J. E. M. Watson

**Affiliations:** 1 Wildlife Conservation Society, Global Conservation Program, Bronx, New York, NY USA; 2 School of Earth and Environmental Sciences, University of Queensland, Brisbane, Australia; 3 Department of Biology, Carleton University, Ottawa, Canada; 4 Wildlife Conservation Society Canada, Toronto, Canada; 5 United Nations Development Programme, One United Nations Plaza, New York, NY USA; 6 World Resources Institute, Washington, DC, USA; 7 Global Earth Observation & Dynamics of Ecosystems Lab, School of Informatics, Computing, and Cyber Systems, Northern Arizona University, Flagstaff, AZ USA; 8 Landscape Biodiversity Lab, Ecology Department, Montana State University, Bozeman, MT USA; 9 Rainforest Foundation Norway, Oslo, Norway; 10 Global Wildlife Conservation, Austin, TX USA; 11 School of Life Sciences, Arizona State University, Tempe, AZ USA; 12 Centre for Tropical Environmental and Sustainability Science, College of Science and Engineering, James Cook University, Cairns, QLD Australia; 13 Environmental Change Institute, School of Geography and the Environment, University of Oxford, Oxford, UK; 14 College of Science and Engineering, James Cook University, Townsville, Queensland Australia; 15 School of Biological Sciences, University of Queensland, St. Lucia, QLD Australia; 16 The Nature Conservancy, Arlington, VA USA; 17 Jet Propulsion Laboratory, California Institute of Technology, Pasadena, CA USA; 18 World Wide Fund for Nature Germany, Space+Science, Berlin, Germany; 19 International Institute of Sustainability, Rio de Janeiro, Brazil; 20 Natural Resource and Environmental Studies Institute, University of Northern British Columbia, Prince George, Canada; 21 International Institute for Applied Systems Analysis, Laxenburg, Austria

**Keywords:** Conservation biology, Ecological modelling, Forest ecology

Correction to: *Nature Communications* 10.1038/s41467-020-19493-3, published online 8 December 2020.

The original version of this Article contained an error in Table 2, in which the total forest area for Protected area category II (third row from the top) was incorrectly stated as 1900 km^2^. The correct value is 1,900,000 km^2^. This has now been corrected in both the PDF and HTML versions of the Article.

