# Supplementary material for: Anthropogenic modification of forests means only 40% of remaining forests have high ecosystem integrity
Source: Nat Commun. 2020 Dec 8;11:5978. doi: 10.1038/s41467-020-19493-3 (PMC7723057; doi:10.1038/s41467-020-19493-3)
Supplement: Supplementary file 1 — Supplementary Information [file 41467_2020_19493_MOESM1_ESM.pdf]

Supplementary Information

Supplementary Note 1: Mapping forest extent

We generated a preliminary base map of global forest extent for the start of 2019 at 30 m resolution by subtracting annual Tree Cover Loss 2001-2018 (with exceptions noted in the next paragraph) from the Global Tree Cover 2000 product [1] using a canopy cover threshold of 20%. This is one of the most widely used tree cover datasets globally, so it has been widely tested in many settings and its strengths and constraints are well understood. It has many advantages, including its high resolution, high accuracy, global coverage, annual time series of tree cover loss and good prospects of sustainability in the coming years. The definition of forest in the source dataset is all woody vegetation taller than 5 m and hence includes naturally regenerated forests as well as tree crops, planted forests, wooded agroforests and urban tree cover. No globally consistent dataset was available that allowed natural and planted tree cover to be consistently distinguished in this study. Therefore, we should be mindful of the many differences between planted and natural tree cover (e.g.[2]).

More than 70% of the tree cover loss shown by the Hansen *et al.* [1] products has been found to be in 10 km pixels where the dominant loss driver is temporary and so tree cover is expected to return above the forest definition threshold within a short period [3]. It is important to take account of this issue as treating all such areas as permanent loss would severely under-estimate current forest cover in many regions. However, no global map of forest cover gain exists for the study period other than the 2000-2012 gain product from Hansen *et al.* [1], so we developed an alternative approach. When removing annual loss shown by the Global Tree Cover Loss product cited above we elected not to remove any loss that was in a 10 km pixel

categorized by Curtis *et al.* [3] as dominated by temporary loss under the categories of fire, shifting cultivation or rotational forestry. This resulted in the adjusted preliminary forest base map. The balance of evidence is that the great majority of such areas would have begun to regenerate and hence qualify as forest by our definition again by 2019 or soon after [3]. The anthropogenically disturbed nature of many of these areas of temporary tree cover loss and recovery is reflected in scoring within the index, because temporary tree cover loss in the categories of shifting cultivation or rotational forestry is treated as an observed pressure. We do not treat tree cover loss through fire as an observed pressure, because fires are often part of natural processes, especially in the boreal zone. This makes our global index conservative as a measure of degradation in these zones, because in some locations fires are anthropogenic in nature.

The adjusted preliminary base map was then resampled to a final base map for 2019 at 300m resolution using a pyramid-by-mode decision rule, with the resulting pixels simply classified as forest or non-forest based on a majority rule. The FLII was calculated for every forest pixel but not for non-forest pixels. GEE performs calculations in WGS84. Supplementary analyses outside GEE were applied using a Mollweide equal-area projection.

Supplementary Note 2: Mapping potential forest configuration

Potential connectivity (PC) is calculated from an estimate of the potential extent of the forest zone taken from Laestadius *et al.* [4], treating areas below 25% crown cover (this was the nearest class to the threshold used in our tree cover dataset of 20%) as non-forest and resampling to 300 m resolution. To minimize false instances of lost connectivity and ensure measures of forest modification are conservative we masked from this data layer areas which

we believe to include a significant proportion of naturally unforested land using selected land-cover categories in ESA ([5]; see Supplementary Table 1). Because these natural non-forest patches are shown in the Hansen *et al.* [1] dataset but not Laestadius *et al.* [4], not excluding such classes would result in an inflated estimate of the loss of connectivity and hence the level of degradation. We have elected to remain conservative in our estimate of modification.

Supplementary Note 3: Mapping observed human pressure

Several recent analyses have developed composite, multi-criteria indices of human pressure to provide assessments of ecosystem condition for the USA [6] or globally [7-9]. Thompson *et al.* [10] set out a framework specific to forest ecosystems that could indicate modification through a balanced mix of available pressure and state variables. We adapted the methodology of Venter et al. [8], informed by the other studies cited, to generate measures of (i) the modification of forest associated with observed human pressure from infrastructure, agriculture and deforestation and (ii) the more diffuse inferred modification effects (e.g. edge effects) whose presence is inferred from proximity to these focal areas of human activity. Edge effects resulting entirely from natural processes are excluded, because they do not represent modification by our definition, although, like many other natural factors, they do also have a role in determining ecosystem benefits.

Infrastructure

We generated the infrastructure (I') data layer by rasterizing the OpenStreetMap data [11] from Feb 2018, using weights for each type of infrastructure as noted in Supplementary Table 3.

The weights were derived from authors' expert opinion and experimentation with weights according to their relative impact on forest condition.

Agriculture

For agriculture (A') we made a global binary composite of the croplands datasets produced by the USGS (Supplementary Table 1) at 30 m resolution, and weighted each cropped pixel at this resolution by the likely intensity of cropping using the global irrigation dataset at 1km resolution (Teluguntla et al, [12]), with values of Irrigation Major = 2, Irrigation Minor = 1.5, Rainfed = 1. The average cropping intensity (including uncropped areas, which score zero) was then calculated across the whole of each 300 m pixel of our final basemap.

Deforestation

For deforestation (H') we made a binary composite of tree cover loss 2001-2018 at 30 m resolution [1], masked out 30 m pixels already classified as agriculture in the preceding step to avoid double-counting, and excluded loss predicted by Curtis *et al.* [3] to be most likely caused by fires, to give a conservative data layer of recent permanent and temporary tree cover loss indicative of human activity in the immediate vicinity. We excluded small clusters of 6 or fewer pixels (0.54 ha) because they may have been natural tree cover loss (e.g. small windthrows) or classification errors. Each 30 m pixel was then weighted by its year of loss, giving higher weight to the most recent loss (2001 = 1, 2002 = 2, etc.). The average 'recentness' of deforestation (including areas not deforested, which score zero) was then calculated across the whole of each 300 m pixel of our base map.

Transformations

The exponential transformations described in the main text were used to convert I', A' and H' to the variables I, A and H respectively.

Supplementary Note 4: Modelling inferred pressures using proximity to observed pressures

Each cell also experiences modification as a result of pressures originating from nearby cells that have observed human pressures, largely through the family of processes known as edge effects [13]. Edge effects are partly a result of the changes relating to biophysical factors (such as humidity, wind, temperature and the increased presence of non-forest species) that accompany the creation of new edges in formerly continuous forest (as exemplified by the carefully controlled studies in tropical forests summarized by Laurance *et al.* [14]). They also result in part from the increased pressure associated with human activities within tropical forest near to edges such as logging [15], anthropogenic fire [16], hunting [17], livestock grazing, pollution, visual and auditory disturbances, etc. These multiple factors are synergistic and so we model them together, notwithstanding regional and local variations in the relative intensity of each one.

We model the inferred effect caused by each nearby source cell as a function of (a) the observed human pressure observed in that source cell and (b) a decline in the intensity of edge effects with distance from the source cell, based on a review of the literature. We then determine the total inferred effect on a given cell by summing the individual effects from all source cells within a certain range.

Two complementary types of inferred effect are modelled and added together. One relates to the diverse, strong, relatively short-range edge effects which decay to near zero over a few kilometers and have the potential to affect most biophysical features of a forest to a greater or lesser extent. The other relates to weaker, longer-range effects such as over-hunting of high-value animals that affect fewer biophysical features of a forest (and so have a much smaller maximum effect on overall integrity) but can nonetheless have detectable effects in locations more than 10 km from the nearest permanent human presence.

The literature on the spatial influence of short-term effects uses a variety of mathematical descriptors, in two broad categories – continuous variables and distance belts. As we wish to model edge effects as a continuous variable we concentrated on studies that have taken a similar approach, and used distance-belt studies as ancillary data.

Chaplin-Kramer *et al.* [18] is a good example of a continuous variable approach, estimating detailed biomass loss curves near tropical forest edges. Because they analyze a key forest condition variable with a very large pantropical dataset we hypothesize that the exponential declines in degradation with distance that they find are likely to be a common pattern and so we use a similar framework for our more general model of degradation. We consider that a model of exponential decay is also a sufficient approximation to the evidence presented by some authors as graphs without an associated mathematical model (e.g., [16,19]) or analyzed using logistic regression (e.g., [20]). In our model we set the exponential decay constant to be broadly consistent with these four studies, resulting in degradation at 1 km inside a forest that is approximately 37% of that at the forest edge, declining to 14% at 2 km and near zero at 3 km. We truncate the distribution at 5 km to minimize computational demands.

Distance-belt studies define the width of a belt within which edge effects are considered to occur, and beyond which forests are considered to be free of edge effect. Belts of 1 km are commonly used (e.g., [13]) but smaller distances may be used for specific parameters (e.g. 300 m for biomass reduction near edges in DRC's primary forests; [21]). Our continuous variable approach is broadly consistent with these studies, with the majority of our modelled degradation within a 1 km belt and little extending beyond 2 km. While most individual edge effects reported in the literature penetrate less than 100-300 m (e.g., [14,22]) most of the effects reported on in these studies relate to the changed natural factors mentioned in an earlier paragraph, and are likely to be dwarfed in both intensity and extent by edge effects relating to spillovers of human activity, so our model emphasizes the spatial distribution of the latter (e.g., [16]). We consider our model of the levels of modification to be conservative.

For the weaker, more widespread long-range effects we use recent large-scale studies of defaunation, which is one of the key long-range pressures and also acts as a proxy for other threats including harvest of high value plants (such as eaglewood *Aquilaria* spp. in tropical Asia), occasional remote fires, pollution associated with artisanal mining, etc. We adopt a simplified version of the distribution used by Peres *et al.* [17] to model hunting around settlements in the Amazon, which sets $2\sigma=12$ km; this is likely conservative compared to evidence for hunting-related declines in forest elephants in central Africa up to 60 km from roads [23] and the extensive declines in large-bodied quarry species in remote areas in many regions modelled by Benitez-Lopez *et al.* [24].

Supplementary Note 5: Exploring limitations in data with an example with infrastructure data in British Columbia, Canada

OpenStreetMap (OSM) represents the most detailed publicly available relevant global dataset but is nonetheless noted to be incomplete, even for one of the most heavily used categories of infrastructure, paved roads [25]. No global assessment is available for the completeness of other categories in the dataset. One of the key categories for forest integrity, unpaved roads used for resource extraction, has been shown to be incomplete over much of insular South-east Asia [26]. In Canada, for example, roads and other linear corridors used to explore, access and extract natural resources (e.g., logging, oil and gas, and minerals) are sometimes missing. Government data for the province of British Columbia (available at https://catalogue.data.gov.bc.ca/dataset/digital-road-atlas-dra-master-partially-attributed-roads) demonstrates, for example, the larger extent and density of regional roads as compared to OSM (Supplementary Figure 1).

Supplementary Note 6: Classification of Forest Landscape Integrity Index scores

In this paper, three illustrative classes were defined, mapped and summarized to give an overview of broad patterns of degradation in the world's forests. Three categories were defined as set out in the Materials and Methods. To determine the approximate levels of the FLII associated with these three categories, benchmark locations were selected in sites that could unambiguously be assigned to one of the categories using the authors' personal knowledge. At each site a single example pixel was selected within a part of the area with relatively uniform scores. The sample points are summarized in Supplementary Table 4; they are widely spread across the world to ensure that the results are not only applicable to a limited region. The scores at these points suggest the following category boundaries:

- High FLII – 9.6-10
- Medium FLII – 6-9.6
- Low FLII – 0-6

Supplementary Note 7: Sensitivity of Forest Landscape Integrity Index scores

To undertake a sensitivity analysis of FLII, we randomly identified 16 000 points dispersed across Earth; from this we found that 1368 points were within forest, which we used for this analysis. For the sensitivity analysis we focused on the infrastructure data given that it is the only variable that that had variable weightings (see Supplementary Table 3). To run the sensitivity analysis we compared the FLII with the following weighting changes applied to the infrastructure data: 1) all infrastructure types were given equal weighting of 10, 2) halved the weightings originally used, and 3) doubled the weightings originally used. We found that there were small changes in the mean FLII and others statistics when the weightings were changed. There was a decrease in the mean when equally weighted or when doubled, and an increase when weightings halved. These changes are quite minor varying a maximum of 0.17, suggesting that adjusting the weightings produced only minor changes in the overall FLII score and the metric is quite robust.

Supplementary Note 8: Verification of Forest Landscape Integrity Index

We anticipate that the FLII is correlated with a broad range of measures related to forest integrity and anthropogenic pressures. These may include metrics related to forest condition (e.g. canopy height, biomass, structural complexity), forest ecosystem state (e.g. species diversity and abundance), and intensity of anthropogenic pressures (e.g. land conversion,

hunting and harvesting, and pollution). The anticipated benefit of FLII is that it provides a single, simple measure that could be used to inform management and policy planning without having to attempt to quantify the full spectrum of metrics related to forest integrity. While it may be possible to do this in some areas, we argue it is infeasible to do so at a global extent and high resolution because of information bias (some areas are much more intensively studied and monitored than others). A strength of FLII is that it is quantified consistently globally, thereby facilitating comparisons among regions and jurisdictions. Moreover, it provides a framework to be adapted for local use where data are available.

Here, we present an example of a validation exercise demonstrating how FLII is correlated with field measurements of forest condition (specifically, canopy height and above-ground biomass) in two countries. In the future, further comparisons of FLII with other measures of forest integrity and in other locations will be made to evaluate the merit of FLII more broadly. We conducted validation analyses across two countries where relevant data were available: Democratic Republic of Congo (DRC), and Myanmar. These countries have recently developed high resolution ecosystem maps to stratify different forest types, and also have appropriate data to test key forest integrity attributes (e.g. canopy height, above ground biomass) [27]. The ecosystem maps used were Shapiro *et al.* [28] for DRC and Murray *et al.* [29] for Myanmar. For DRC we correlated FLII with ~415,000 samples of mean canopy height and estimated above ground biomass, based on field samples and lidar data from Xu *et al.* [30]. We analysed these samples at the country level, and also stratified samples by forest ecosystem type. For Myanmar we generated 5000 random points for each forest ecosystem, and then extracted FLII, tree canopy height and tree canopy cover for each point using data from Potapov *et al.* [31]. We then analysed data at the country level, and used the hierarchical nature of the Myanmar ecosystem data to assess forest ecosystems individually, and also grouped

ecosystems into five broader functional groups see Murray *et al.* [32]. For all analyses we measured correlation using Kendall's T coefficient, because our data did not satisfy the assumption of normality required to conduct a Pearson correlation test. Kendall's T coefficient ranges from -1 – 1, where ±1 indicates a perfect degree of correlation, and 0 indicates no correlation.

For DRC as a whole we found a positive and significant correlation ($p < 0.05$) between FLII and both above ground biomass and mean canopy height (Supplementary Figure 3). We also found a positive and significant correlation ($p < 0.05$) between FLII and above ground biomass for 31 of 37 ecosystem types (Supplementary Figure 4), and the same result for mean tree canopy height (Supplementary Figure 5). For Myanmar as a whole we found a positive and significant correlation ($p < 0.05$) between FLII and both tree canopy height and tree canopy cover (Supplementary Figure 6). We also found a positive and significant correlation ($p < 0.05$) between FLII and tree canopy height across 29 of 30 ecosystem types (Supplementary Figure 7), and the same result for tree canopy cover (Supplementary Figure 8). For all ecosystem functional groups ($n=5$) in Myanmar we found similar trends to ecosystems, with strong positive correlations between FLII and tree canopy height and tree canopy cover for every group (Supplementary Figure 9-11). Overall, we found a positive and significant correlation ($p<0.05$) across 134 out of 148 tests.

Supplementary Table 1. The datasets used to develop the Forest Landscape Integrity Index. The factor column indicates the component of the index the dataset was used in.

Dataset	Factor	Sources	Spatial resolution	Time period
**Tree cover and tree cover loss**	Forest extent, connectivity, observed and inferred pressures	Global Forest Cover datasets; Hansen *et al.* [1]; updates to 2018 available on-line from: http://earthenginepartners.appspot.com/science-2013-global-forest.	30m	2000-2018
**Major tree cover loss driver**	Forest extent, observed and inferred pressures, connectivity	Curtis *et al.* [3]	10km	2000-2018
**Landover and ocean extent**	Forest extent	Lamarche *et al.* [33]	150m	2000-2012
**Potential forest cover**	Connectivity	Laestadius *et al.* [4]	1km	2010
**Natural non-forest areas within extent of potential forest**	Connectivity	ESA-CCI Land Cover dataset; ESA [5]	300m	2015
**Infrastructure**	Observed and inferred pressures	Open Street Map (selected elements) as of 2018; OpenStreetMap contributors [11]	300m (Derived from vectors)	2004-2018
**Cropland**	Observed and inferred pressures	GFSAD 2015 Cropland Extent; Gumma *et al.* [34], Massey *et al.* [35], Oliphant *et al.* [36], Phalke *et al.* [37], Teluguntla *et al.* [38], Xiong *et al.* [39] and Zhong *et al.* [40]	30m	2015
**Cropping intensity (irrigation)**	Observed and inferred pressures	GFSAD 2010 Cropland Mask; Teluguntla *et al.* [12]	1km	2010
**Water surface**	Observed and inferred pressures	JRC Global Surface Water Occurrence (all classes with >75% occurrence); Pekel *et al.* [41]	30m	1984-2019

Supplementary Table 2. Classes in ESA-CCI dataset excluded from our potential forest cover layer because they overlap extensively with potential forest cover mapped by Laestadius *et al.* [4] but contain significant areas of natural non forest

**Legend code**	**Class name**
60	Treecover, broadleaved, deciduous, closed to open, >15%
100	Mosaic tree and shrub (>50%]/ Herbaceous cover (<50%)
120	Shrubland
121	Evergreen shrubland
122	Deciduous shrubland
130	Grassland
140	Lichens and mosses
150	Sparse vegetation (tree, shrub, herbaceous cover) (<15%)
152	Sparse shrub (<15%)
180	Shrub or herbaceous cover, flooded, fresh/saline/brackish water
200	Bare areas
201	Consolidated bare areas
202	Unconsolidated bare areas
220	Permanent snow and ice

Supplementary Table 3. Weightings used for Open Street Map (OSM) to combine into the Infrastructure data layer.

OSM Category	OSM Subcategory	Weighting applied for FPI
**Aeroway**	Apron / Helipad / Runway / Taxiway	8
	Hangar / Terminal	4
	Aerodrome / Heliport / Spaceport	3
**Amenity / Landuse / Man-made object**	Fuel station / Gasometer / Petroleum well / Pipeline / Adit / Mineshaft / Quarry / Landfill / Sanitary dump station / Wastewater plant	15
	Chimney	10
	Industrial	8
	Basin / Covered Reservoir / Pumping station / Water tower / Water well / Water works / Watermill	7
	Silo / Storage tank / Works	6
	Aerialway / Beacon / Lighthouse / Breakwater / Dyke / Embankment / Groyne / Pier / Communications tower / Mast / Observatory / Tower / Telescope	5
	Salt pond	4
	Alpine hut / Beach resort / Camp site / Cemetery / Golf course / Marina / Pitch / Village green / Wilderness hut	3
**Barrier**	City wall / Retaining wall / Wall	5
	Ditch / Snow fence / Snow net	3
	Hedge	2
**Road**	Motorway / Motorway link / Raceway	15
	Trunk / Trunk link	11
	Primary / Primary link	9
	Secondary / Secondary link	7
	Tertiary / Tertiary link	6
	Bus guideway / Service	5
	Living street / Mini roundabout / Residential / Turning circle / Unclassified / Unknown/ Elevator / Rest area	4
	Escape / Track	3
	Bridleway / Cycleway/ Footway / Path / Pedestrian / Steps	2
**Military**	Nuclear explosion site	30
	Danger area / Range / Trench	15
	Ammunition / Barracks / Bunker / Checkpoint	7
	Airfield / Military-owned land / Naval base / Training area	3
**Power**	Plant/generator - coal	20
	Plant/generator - oil	15
	Plant/generator – gas/ Plant/generator - bio / waste	10
	Plant/generator – hydro; nuclear; other / Line, Substation	7
	Plant/generator - solar / Heliostat / wind / Windmill	5
	Cable	3
**Railway**	Funicular / Preserved / Rail / Monorail / Subway	10
	Light rail / Miniature / Narrow gauge/ Tram	7
	Station	5
	Halt / Platform	4
	Abandoned / Disused	2
**Waterway**	Dam / Lock gate	20
	Canal	13
	Ditch/ Drain / Weir	3

Supplementary Table 4. Points assessed to determine category boundaries for classifying the FHI into high, medium and low classes.

Category	Code	Point description	Country	Point Score
**High**	103	Interior of Lopé National Park	Gabon	10.000
**High**	106	Interior of Taï National Park	Cote d'Ivoire	10.000
**High**	108	Interior of Pacaya-Samiria National Reserve	Peru	10.000
**High**	109	Interior of Central Suriname Nature Reserve	Suriname	10.000
**High**	116	Interior of Liard River area	Canada	10.000
**High**	101	Interior of Okapi Faunal Reserve	DRC	9.997
**High**	104	Interior of Nyungwe National Park	Rwanda	9.992
**High**	111	Interior of Rio Platano Biosphere Reserve	Honduras	9.990
**High**	102	Interior of Odzala National Park	RoC	9.974
**High**	117	Interior of Wells Gray Provincial Park	Canada	9.972
**High**	119	Interior of Øvre Pasvik National Park	Norway	9.944
**High**	115	Interior of Tasmania Wilderness World Heritage Area	Australia	9.918
**High**	107	Interior of Marojejy National Park	Madagascar	9.910
**High**	112	Interior of Khao Yai National Park	Thailand	9.908
**High**	105	Interior of Niassa Special Reserve	Mozambiuque	9.819
**High**	110	Interior of Maya Biosphere Reserve	Guatemala	9.798
**High**	114	Interior of Batang Ai National Park	Malaysia	9.756
**High**	118	Interior of Quetico Provincial Park	Canada	9.750
**High**	113	Interior of Sundarbans National Park	Bangladesh	9.606
**Medium**	215	Interior of Bialowieża National Park	Poland	9.086
**Medium**	208	Interior of Mabira Central Forest Reserve	Uganda	9.067
**Medium**	211	Area of selective logging	Gabon	8.840
**Medium**	219	Near main tourism corridor, Mt Myohyang National Park	DPR Korea	8.762
**Medium**	203	Interior of Phnom Kulen Wildlife Sanctuary	Cambodia	8.710
**Medium**	210	Area of selective logging	Guyana	8.364
**Medium**	202	Interior of Dong Hua Sao National Protected Area	Lao PDR	8.078
**Medium**	212	Area of selective logging	DRC	7.981
**Medium**	206	Interior of Manga Forest Reserve	Tanzania	7.960
**Medium**	207	Near margin of Nyungwe National Park	Rwanda	7.938
**Medium**	204	South part of Nagarahole National Park	India	7.759
**Medium**	213	Area of selective logging	Cameroon	7.379
**Medium**	201	Tat Leuk, Phou Khaokhoay National Protected Area	Lao PDR	7.251
**Medium**	216	Interior of Loch Garten Nature Reserve	UK	7.146
**Medium**	209	Area of selective logging	Congo	6.734
**Medium**	217	Tourism area, Lamington National Park	Australia	6.729
**Medium**	214	Lowlands of Guanacaste National Park	Costa Rica	6.719
**Medium**	218	Near margin of Sepilok Forest Reserve	Malaysia	6.353
**Medium**	205	Interior of Similajau National Park	Malaysia	6.130
**Low**	305	Dong Nathat	Lao PDR	5.638
**Low**	317	Foothills of Mt Makiling	Philippines	5.395
**Low**	310	Suburban woodlot, Dobbs Ferry	USA	4.710
**Low**	309	Jozani Forest Reserve	Tanzania	4.680
**Low**	316	Foothills of Mt Canlaon	Philippines	4.597
**Low**	320	Forest fragment near Paramaribo	Suriname	4.566
**Low**	302	Central Park, New York	USA	3.575
**Low**	301	Bagley Wood, Oxford	UK	3.525
**Low**	307	Boeng Yeak Lom Protected Area	Cambodia	3.323
**Low**	304	Angkor Thom	Cambodia	3.122
**Low**	315	Forest in rural complex, Mambasa area	DRC	2.689
**Low**	312	Woodland in Beaumont area	USA	2.581
**Low**	318	Swidden near Andoung Kraloeng village	Cambodia	2.304

**Low**	319	Forest mosaic near Kaev Seima village	Cambodia	2.187
**Low**	303	Thetford Forest	UK	2.082
**Low**	313	Woodland in Augusta area	USA	0.686
**Low**	314	Woodland in Emporia area	USA	0.589
**Low**	311	River Park, Chicago	USA	0.566
**Low**	306	Houei Nhang Forest Reserve	Lao PDR	0.000
**Low**	308	Pugu Forest Reserve	Tanzania	0.000

Supplementary Table 5. Mean Forest Landscape Integrity Index scores and areas for forest integrity categories by country.

Country	Mean FLII	Low integrity (km^2)	Medium integrity (km^2)	High integrity (km^2)	Total forest area (km^2)
**Afghanistan**	8.85	90	1,475	977	2,542
**Albania**	6.77	2,426	5,256	122	7,805
**Algeria**	5.22	7,418	6,044	81	13,543
**Andorra**	4.45	170	49	0	219
**Angola**	8.35	105,487	284,054	315,895	705,436
**Antigua and Barbuda**	4.72	114	92	0	206
**Argentina**	7.21	98,249	189,966	72,557	360,772
**Armenia**	5.46	1,894	1,681	3	3,577
**Australia**	7.22	117,672	239,624	103,852	461,148
**Austria**	3.55	36,666	12,422	21	49,109
**Azerbaijan**	6.55	4,820	7,189	1,534	13,543
**Bahamas**	7.35	741	1,935	399	3,075
**Bangladesh**	5.45	10,013	7,251	1,947	19,211
**Belarus**	3.63	77,870	20,847	91	98,808
**Belgium**	1.36	8,803	297	0	9,099
**Belize**	6.15	7,004	7,957	2,744	17,705
**Benin**	5.86	4,724	3,698	1,769	10,191
**Bhutan**	8.85	1,620	16,769	10,140	28,529
**Bolivia**	8.47	78,745	280,532	272,007	631,284
**Bosnia and Herzegovina**	5.99	13,387	17,031	574	30,993
**Botswana**	9.13	13	187	372	572
**Brazil**	7.52	1,374,902	1,354,961	2,338,101	5,067,963
**Brunei Darussalam**	7.71	1,102	2,842	1,498	5,442
**Bulgaria**	6.09	18,884	26,325	847	46,057
**Burundi**	4.5	6,882	3,841	46	10,769
**Cabo Verde**	6.37	27	38	0	65
**Cambodia**	6.31	30,143	31,939	16,349	78,431
**Cameroon**	8	66,191	181,336	119,263	366,789
**Canada**	8.99	480,206	1,027,386	2,968,268	4,475,860
**Central African Republic**	9.28	30,161	139,350	379,097	548,608
**Chad**	6.18	5,261	6,016	1,910	13,187
**Chile**	7.37	56,849	41,971	93,537	192,357
**China**	7.14	533,800	974,431	301,051	1,809,282
**Colombia**	8.26	150,737	272,442	428,320	851,499
**Comoros**	7.69	284	1,149	82	1,515

**Congo**	8.89	24,512	124,215	158,184	306,911
**Congo DRC**	7.56	533,118	935,508	727,983	2,196,608
**Costa Rica**	4.65	27,164	12,838	4,164	44,167
**Cote d'Ivoire**	3.64	158,010	41,005	7,288	206,303
**Croatia**	4.92	15,732	10,522	379	26,633
**Cuba**	5.4	22,605	18,460	1,632	42,697
**Cyprus**	7.06	388	1,026	18	1,432
**Czechia**	1.71	32,161	1,611	0	33,772
**Denmark**	0.5	5,756	31	0	5,787
**Dominica**	1.06	531	2	0	533
**Dominican Republic**	4.19	19,890	9,364	518	29,772
**Ecuador**	7.66	48,822	77,585	73,492	199,900
**Egypt**	0.56	4,772	218	69	5,059
**El Salvador**	4.05	8,837	2,947	0	11,784
**Equatorial Guinea**	7.99	3,982	17,595	5,007	26,585
**Estonia**	3.05	24,473	4,832	52	29,358
**Ethiopia**	7.16	52,652	84,430	44,397	181,479
**Fiji**	8.35	1,753	10,802	3,594	16,148
**Finland**	5.08	144,310	83,572	9,294	237,176
**France**	4.52	161,987	49,496	74,121	285,604
**Gabon**	9.07	11,780	118,348	120,852	250,979
**Gambia**	4.56	181	85	0	266
**Georgia**	7.79	6,982	17,803	9,784	34,570
**Germany**	2.28	122,168	11,307	0	133,475
**Ghana**	4.53	57,519	28,901	2,160	88,580
**Greece**	6.6	14,548	27,833	1,078	43,459
**Grenada**	4.22	221	86	0	308
**Guatemala**	3.85	58,572	18,764	5,592	82,928
**Guinea**	4.9	81,702	54,877	2,895	139,475
**Guinea-Bissau**	5.7	9,274	8,702	855	18,831
**Guyana**	9.58	4,162	40,817	147,413	192,391
**Haiti**	4.01	7,116	2,831	12	9,959
**Honduras**	4.48	57,899	23,802	3,692	85,392
**Hungary**	2.25	18,729	2,047	0	20,776
**India**	7.09	117,992	254,792	54,428	427,211
**Indonesia**	6.6	535,370	509,018	431,973	1,476,361
**Iran**	7.67	3,361	12,930	2,162	18,453
**Iraq**	3.59	104	9	0	113
**Ireland**	0.92	5,283	96	0	5,378
**Israel**	4.14	170	85	0	255
**Italy**	3.65	79,403	26,858	25	106,286
**Jamaica**	5.01	5,362	3,249	158	8,770

**Japan**	5.8	135,783	133,480	16,005	285,268
**Jordan**	2.79	12	0	0	12
**Kazakhstan**	8.23	6,068	18,926	15,294	40,288
**Kenya**	4.2	28,427	13,558	4,702	46,686
**Kosovo**	5.19	2,628	1,775	47	4,450
**Kyrgyzstan**	8.86	329	2,819	2,761	5,909
**Laos**	5.59	92,986	80,564	19,252	192,801
**Latvia**	2.09	38,164	2,137	0	40,301
**Lebanon**	3.76	541	115	0	656
**Lesotho**	7.4	1	4	0	5
**Liberia**	4.79	51,975	31,162	11,025	94,163
**Libya**	4.85	15	2	0	17
**Liechtenstein**	4.5	59	42	0	101
**Lithuania**	1.62	24,554	930	0	25,484
**Luxembourg**	1.12	1,170	0	0	1,170
**Macedonia**	7.42	2,034	7,090	459	9,583
**Madagascar**	4.63	120,340	66,584	11,922	198,846
**Malawi**	5.74	12,514	12,167	2,396	27,078
**Malaysia**	5.01	130,825	91,957	21,499	244,281
**Mali**	7.16	451	996	140	1,586
**Mauritius**	5.46	567	478	0	1,045
**Mexico**	6.82	193,908	280,445	121,842	596,195
**Micronesia**	7.55	8	35	0	43
**Moldova**	2.2	3,113	202	0	3,315
**Mongolia**	9.36	520	11,915	27,407	39,841
**Montenegro**	6.41	2,949	4,778	82	7,809
**Morocco**	6.74	2,260	4,076	451	6,787
**Mozambique**	6.93	150,665	189,362	115,379	455,406
**Myanmar**	7.18	129,745	220,188	96,924	446,857
**Namibia**	8.43	5	13	17	36
**Nepal**	7.23	13,785	41,992	3,760	59,538
**Netherlands**	0.6	5,250	72	0	5,322
**New Zealand**	7.12	34,503	44,155	35,334	113,992
**Nicaragua**	3.63	65,356	17,646	4,858	87,860
**Nigeria**	6.2	64,621	65,355	24,307	154,283
**North Korea**	8.02	8,374	40,156	8,410	56,939
**Norway**	6.98	39,343	67,383	16,627	123,352
**Pakistan**	7.42	2,090	7,859	1,139	11,088
**Palau**	8.09	45	333	9	387
**Panama**	6.37	25,420	21,310	14,605	61,336
**Papua New Guinea**	8.84	37,294	183,415	216,355	437,064
**Paraguay**	6.39	78,538	102,626	29,877	211,041

**Peru**	8.86	85,793	190,547	509,720	786,061
**Philippines**	5.91	91,820	100,831	8,393	201,044
**Poland**	2.24	101,886	7,103	0	108,989
**Portugal**	0.82	25,966	553	0	26,519
**Romania**	5.95	38,395	48,394	607	87,395
**Russian Federation**	9.02	739,484	2,245,281	5,137,079	8,121,843
**Rwanda**	3.85	5,665	2,170	619	8,454
**Saint Kitts and Nevis**	4.55	95	50	0	145
**Saint Lucia**	6.17	235	316	0	551
**Saint Vincent and the Grenadines**	6.95	91	221	0	312
**San Marino**	0.01	7	0	0	7
**Sao Tome and Principe**	6.64	31	140	0	171
**Senegal**	7.11	847	2,456	162	3,465
**Serbia**	5.29	17,513	14,112	516	32,141
**Seychelles**	10	0	0	68	68
**Sierra Leone**	2.76	52,512	11,858	640	65,010
**Singapore**	1.11	170	2	0	172
**Slovakia**	4.34	17,615	8,165	0	25,781
**Slovenia**	3.78	11,065	3,791	0	14,856
**Solomon Islands**	7.19	6,871	15,310	3,149	25,329
**Somalia**	7.16	347	1,384	46	1,777
**South Africa**	4.94	45,489	34,968	3,196	83,653
**South Korea**	6.02	25,060	32,009	888	57,956
**South Sudan**	9.45	5,083	59,389	146,218	210,691
**Spain**	4.23	82,770	46,013	133	128,916
**Sri Lanka**	5.83	20,541	22,390	1,613	44,544
**Sudan**	9.8	1	72	495	569
**Suriname**	9.39	6,796	25,031	107,954	139,781
**Swaziland**	4.21	5,054	2,501	14	7,569
**Sweden**	5.35	174,415	109,779	23,494	307,687
**Switzerland**	3.53	13,636	4,412	10	18,058
**Syria**	3.64	841	282	0	1,123
**Taiwan**	6.38	8,786	14,547	1,453	24,786
**Tajikistan**	8.65	34	137	130	301
**Tanzania**	7.13	123,997	159,712	122,812	406,521
**Thailand**	6	86,276	89,326	33,612	209,214
**Timor-Leste**	7.11	1,783	7,008	47	8,838
**Togo**	5.88	5,064	4,522	1,076	10,662
**Trinidad and Tobago**	6.62	1,478	2,176	418	4,072
**Tunisia**	5.14	1,354	987	0	2,340
**Turkey**	6.39	43,043	68,243	3,516	114,801

**Turkmenistan**	6.31	5	33	0	37
**Uganda**	4.36	77,303	36,381	7,507	121,190
**Ukraine**	3.3	89,540	20,183	176	109,900
**United Kingdom**	1.65	29,149	2,917	35	32,101
**United States**	6.65	1,328,079	1,144,693	658,645	3,131,417
**Uruguay**	3.61	11,793	3,998	0	15,791
**Uzbekistan**	6.77	214	227	199	640
**Vanuatu**	8.82	734	5,322	4,448	10,504
**Venezuela**	8.78	64,650	170,792	351,112	586,554
**Vietnam**	5.35	82,551	75,353	9,588	167,492
**Zambia**	7.5	96,969	164,376	110,822	372,167
**Zimbabwe**	6.31	9,450	14,417	1,644	25,511

Supplementary Table 6. Mean Forest Landscape Integrity Index (FLII) scores for provinces of Democratic Republic of Congo (DRC), Indonesia and Canada.

**DRC**		**Indonesia**		**Canada**	
**Province**	**Mean FLII**	**Province**	**Mean FLII**	**Province**	**Mean FLII**
Lualaba	8.57	Papua	9.34	Northwest Territories	9.90
Tshuapa	8.55	West Papua	9.00	Yukon	9.86
Tshopo	8.39	Kalimantan Utara	8.52	Newfoundland and Labrador	9.66
Bas-Uélé	8.38	Maluku	8.03	Nunavut	9.65
Équateur	8.37	Maluku Utara	7.41	Manitoba	9.58
Haut-Lomami	8.29	Nusa Tenggara Barat	6.86	Saskatchewan	9.40
Tanganyika	8.24	Aceh	6.83	Ontario	8.94
Nord-Ubangi	8.19	Nusa Tenggara Timur	6.80	Québec	8.80
Haut-Katanga	8.05	Gorontalo	6.60	Alberta	8.46
Kwango	7.83	Sulawesi Utara	6.58	British Columbia	8.22
Maï-Ndombe	7.58	Sulawesi Tengah	6.54	Nova Scotia	6.07
Haut-Uélé	7.46	Kalimantan Timur	6.42	New Brunswick	5.15
Maniema	7.44	Sulawesi Barat	6.31	Prince Edward Island	2.74
Sankuru	7.34	Sumatera Barat	6.20		
Lomami	7.20	Sulawesi Tenggara	5.99		
Kasaï	7.11	Kalimantan Tengah	5.84		
Ituri	6.70	Sulawesi Selatan	5.63		
Mongala	6.23	Banten	4.97		
Nord-Kivu	6.22	Bengkulu	4.94		
Sud-Kivu	6.20	Sumatera Utara	4.89		
Kasaï-Central	5.95	Kalimantan Barat	4.87		
Sud-Ubangi	5.93	Kepulauan Riau	4.86		

Kwilu	5.65	Jawa Barat	4.76		
Kinshasa	4.75	Lampung	4.73		
Kasaï-Oriental	4.13	Jawa Tengah	4.59		
Kongo-Central	3.95	Bali	4.43		
		Jawa Timur	4.40		
		Jambi	4.01		
		Riau	3.92		
		Kalimantan Selatan	3.24		
		Sumatera Selatan	2.86		
		Yogyakarta	2.83		

Supplementary Table 7. Sensitivity analysis varying the weightings within the infrastructure data

	Mean	1st quartile	Median	3rd quartile	Standard Deviation
**Original**	7.49	5.59	9.04	9.93	3.06
**Equal weighted**	7.32	5.26	8.95	9.92	3.20
**Weighting halved**	7.64	6.06	9.12	9.94	2.94
**Weighting doubled**	7.36	5.30	8.96	9.93	3.17

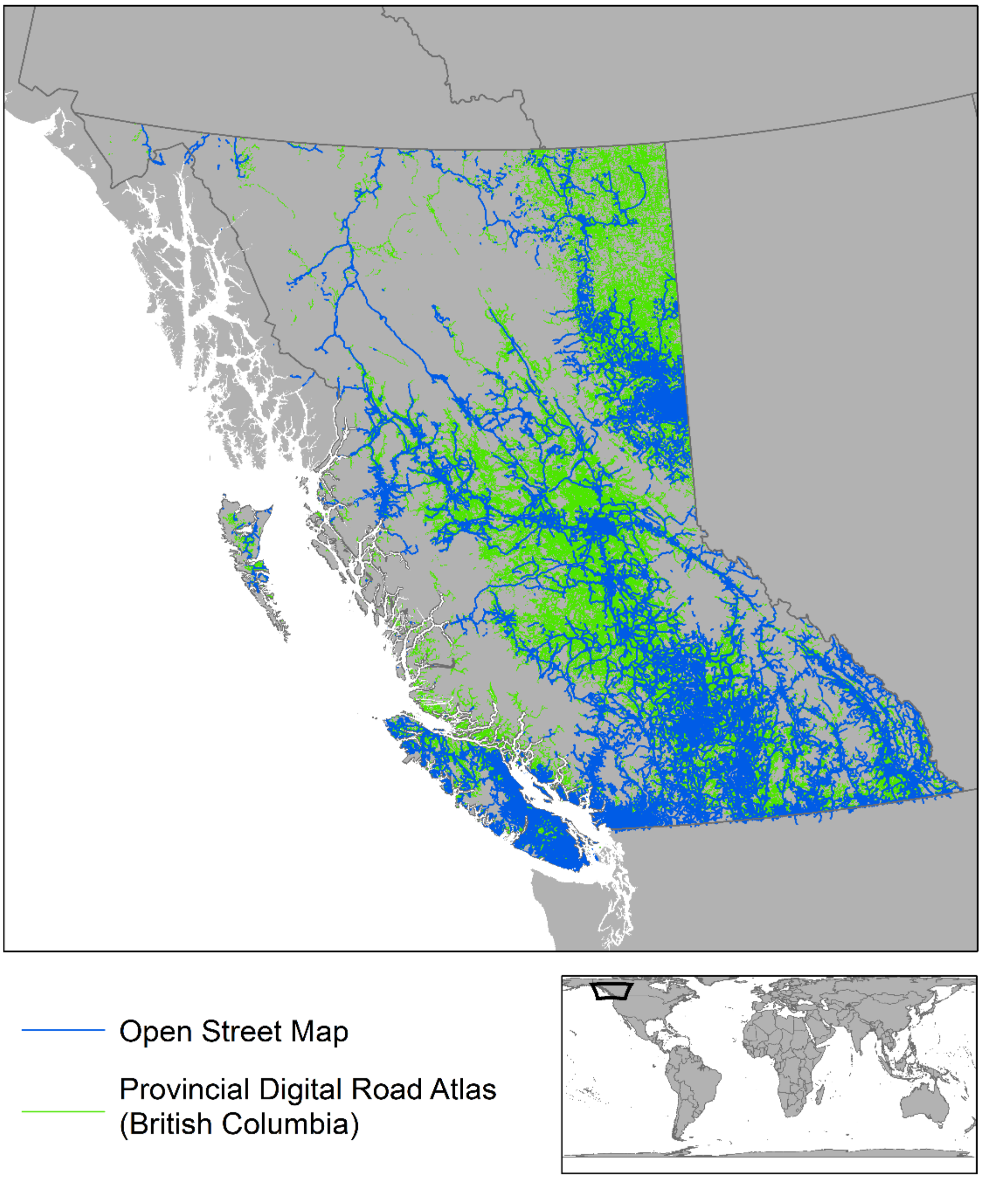

Supplementary Figure 1. A map overlaying the Open Street Maps data (blue) and provincial government data (green) for roads and other linear infrastructure associated with resource access.

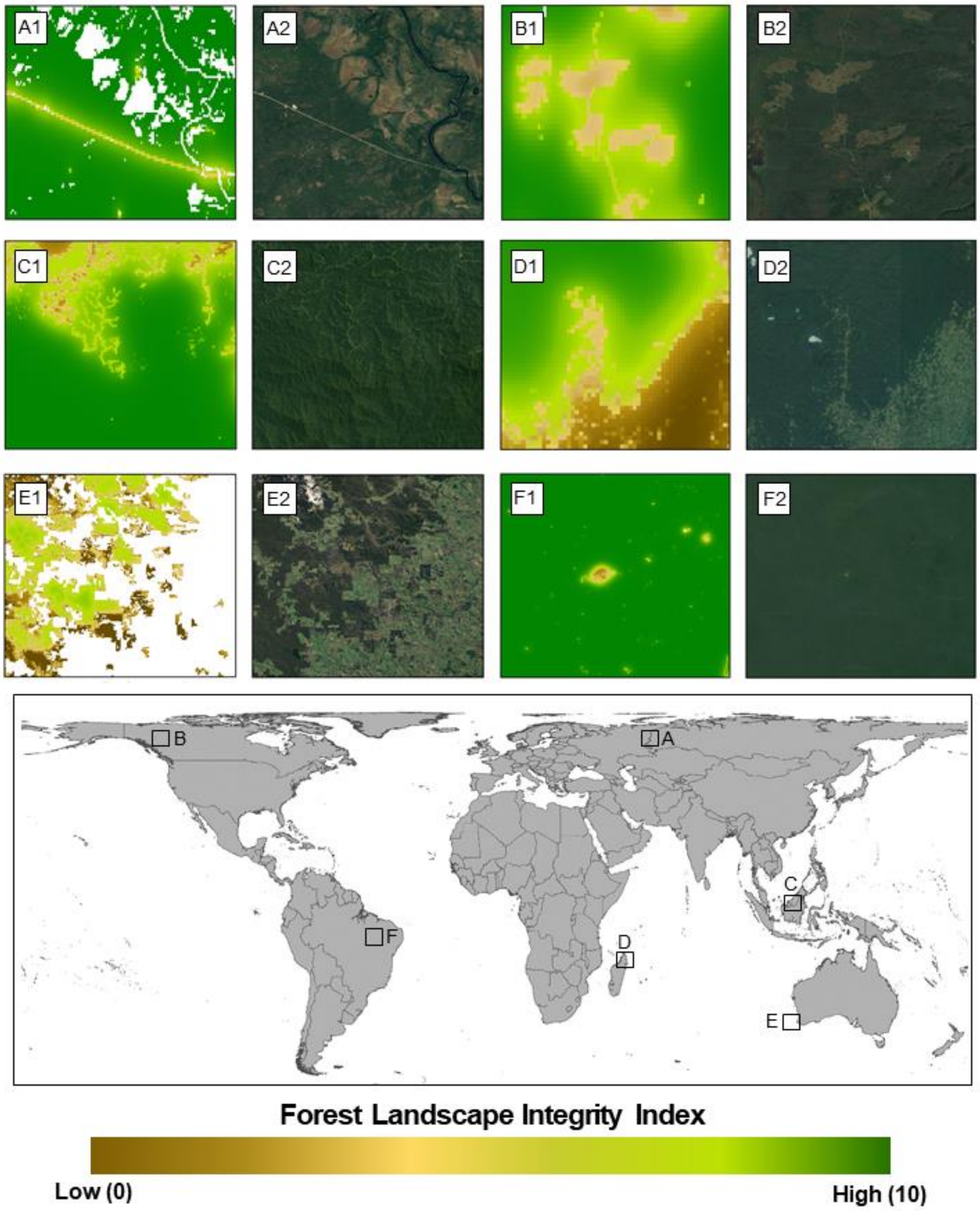

Supplementary Figure 2. A global map of Forest Landscape Integrity for 2019. Highlighted regions show **A.** A remote road in Russia, **B.** Clearcut logging in Canada, **C.** Selective logging in Borneo, **D.** Swidden agriculture in Madagascar, **E.** Forest fragmentation in Western Australia, **F.** Remote settlements in the Brazilian Amazon.

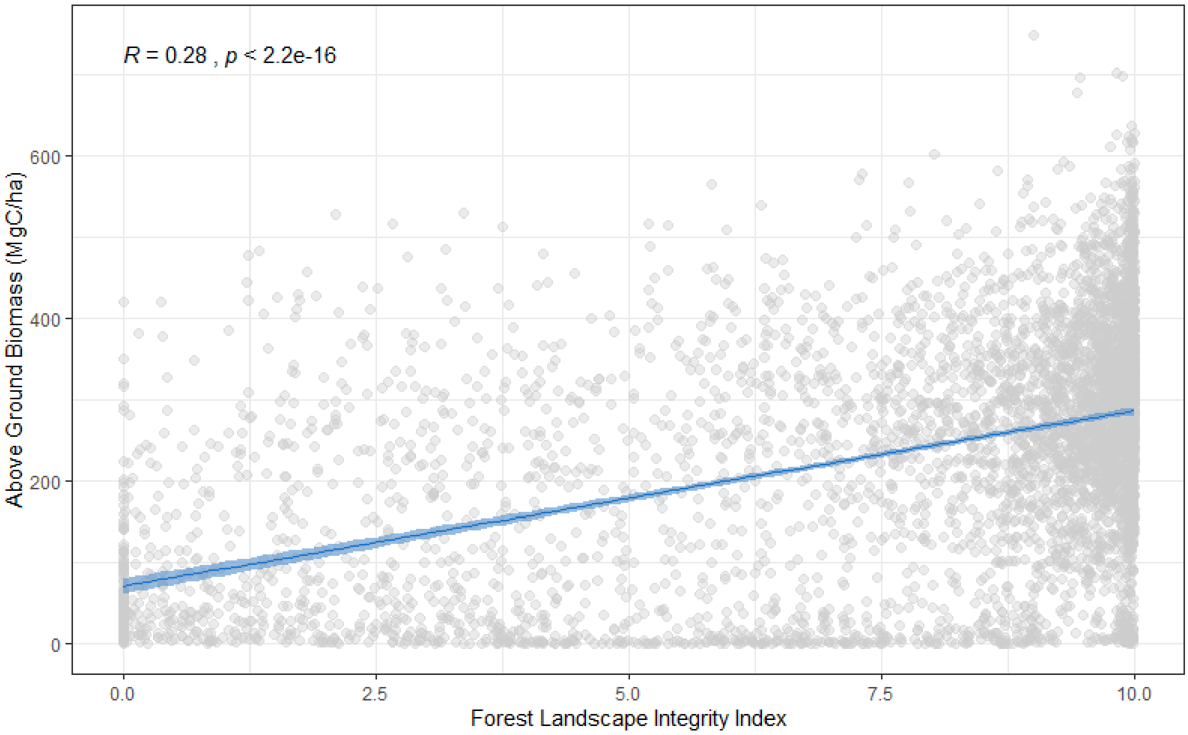

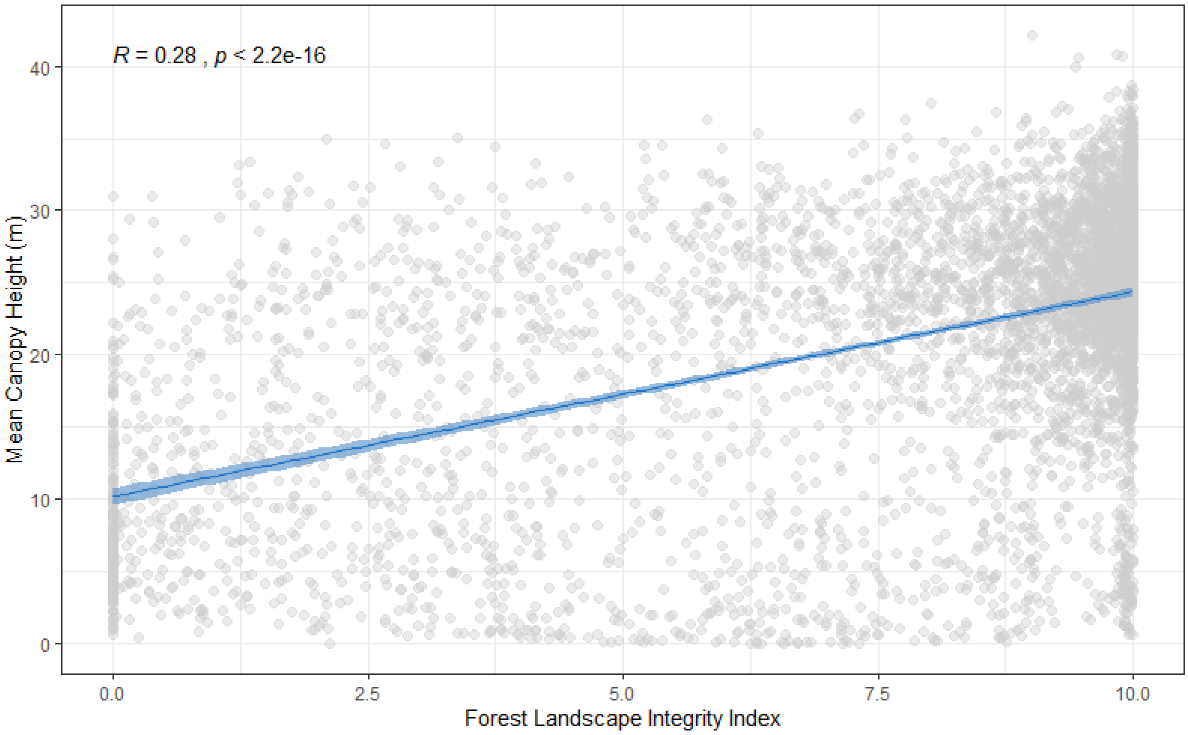

Supplementary Figure 3. Correlation between Forest Landscape Integrity Index and above ground biomass (above) and mean canopy height (below) within Democratic Republic of Congo. N = 5000 independent samples for each forest ecosystem. Correlation was measured using a two-sided Kendall's T coefficient (P < 2.2-16 for both above ground biomass and

mean canopy height). Coloured lines are linear model fits with 95% confidence intervals (shaded regions).

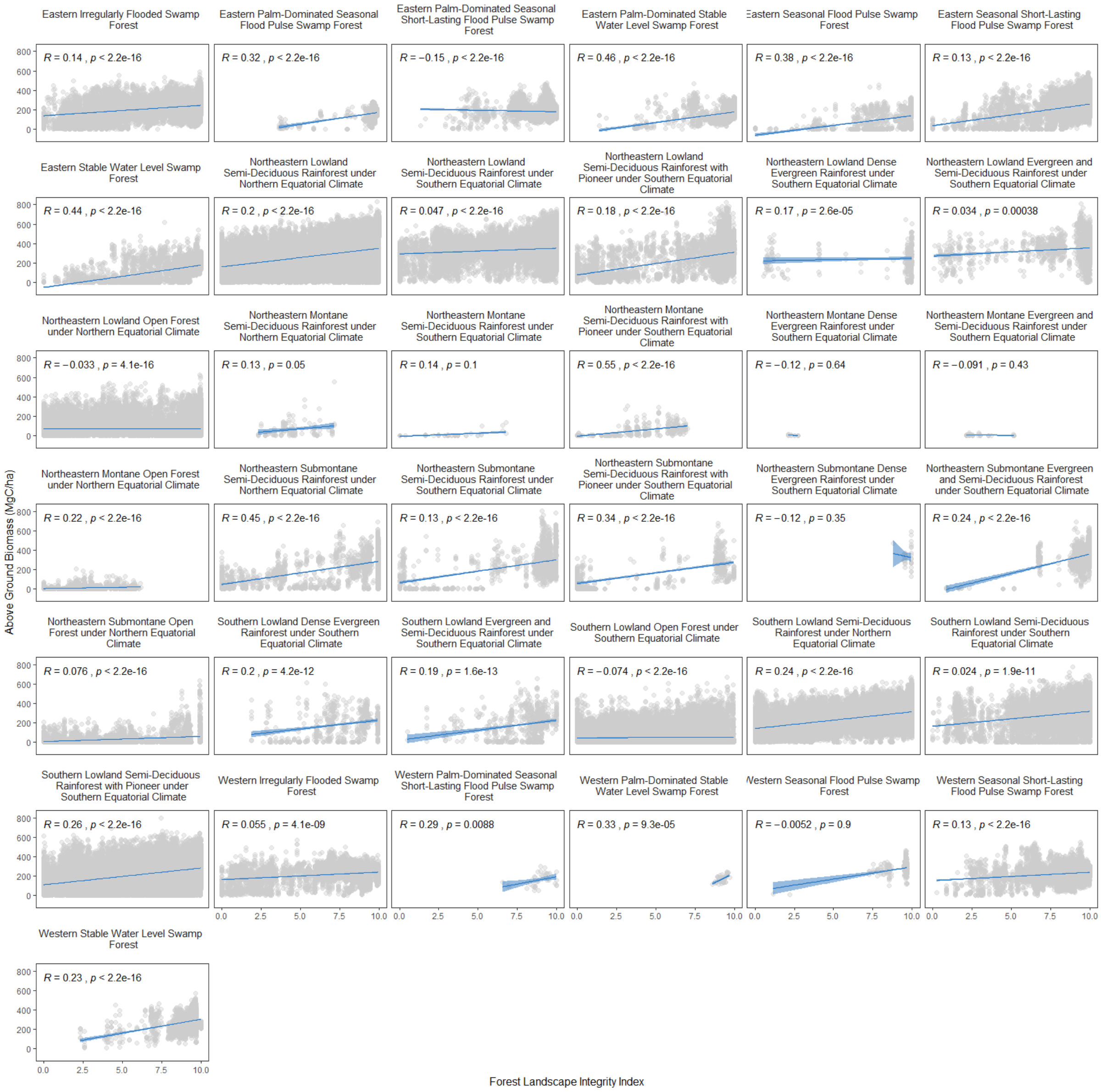

Supplementary Figure 4. Correlation between Forest Landscape Integrity Index and above ground biomass for forest ecosystems within the Democratic Republic of Congo. N = 2500 independent samples for each forest ecosystem. Correlation was measured using a two-sided Kendall's T coefficient. Coloured lines are linear model fits with 95% confidence intervals (shaded regions).

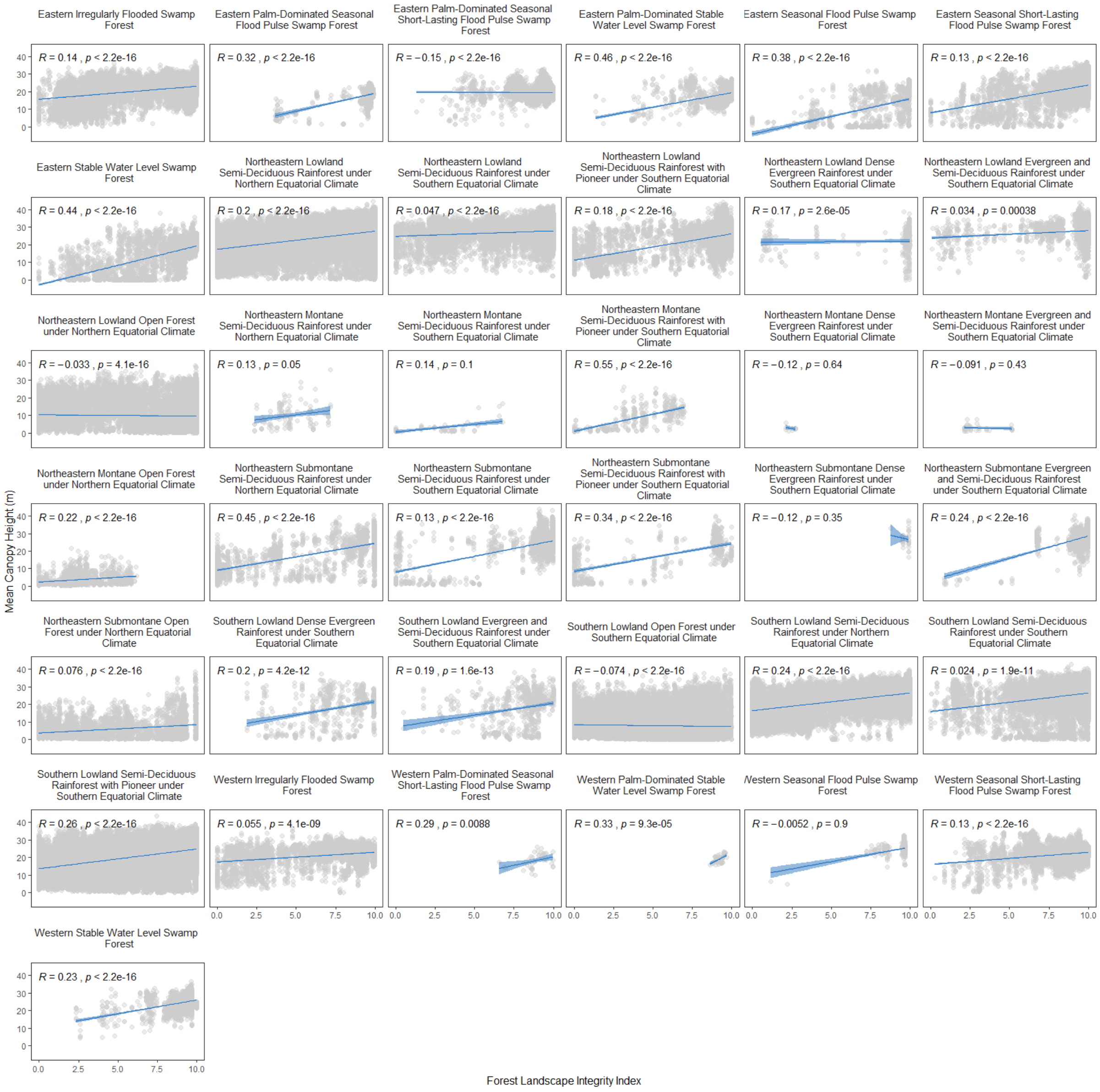

Supplementary Figure 5. Correlation between Forest Landscape Integrity Index and mean canopy height for forest ecosystems in the Democratic Republic of Congo. N = 2500 independent samples for each forest ecosystem. Correlation was measured using a two-sided Kendall's T coefficient. Coloured lines are linear model fits with 95% confidence intervals (shaded regions)..

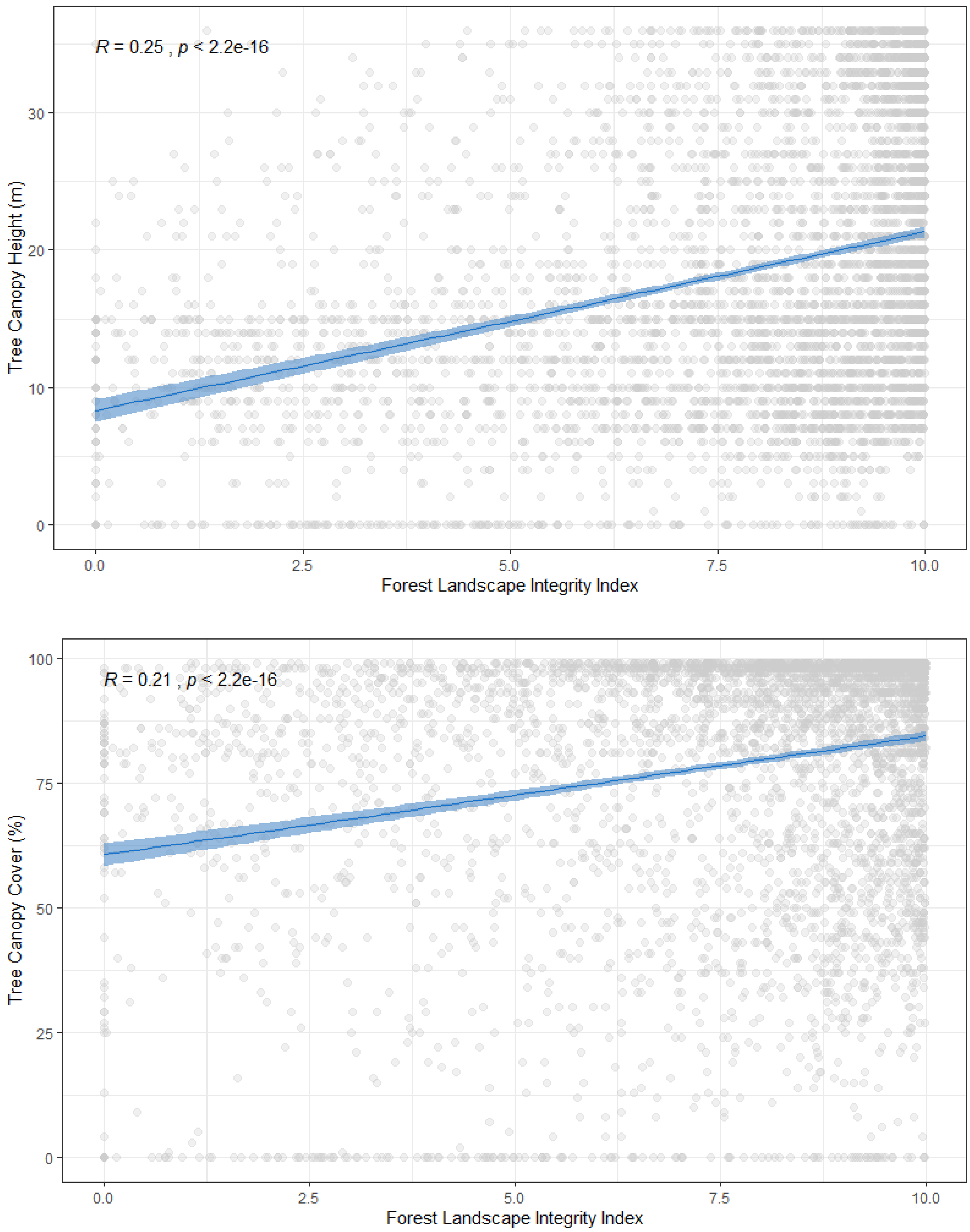

Supplementary Figure 6. Correlation between Forest Landscape Integrity Index and tree canopy height (above) and tree canopy cover (below) within Myanmar. N = 5000 independent samples. Correlation was measured using a two-sided Kendall's T coefficient (P

< 2.2-16 for both above ground biomass and mean canopy height). Coloured lines are linear model fits with 95% confidence intervals (shaded regions).

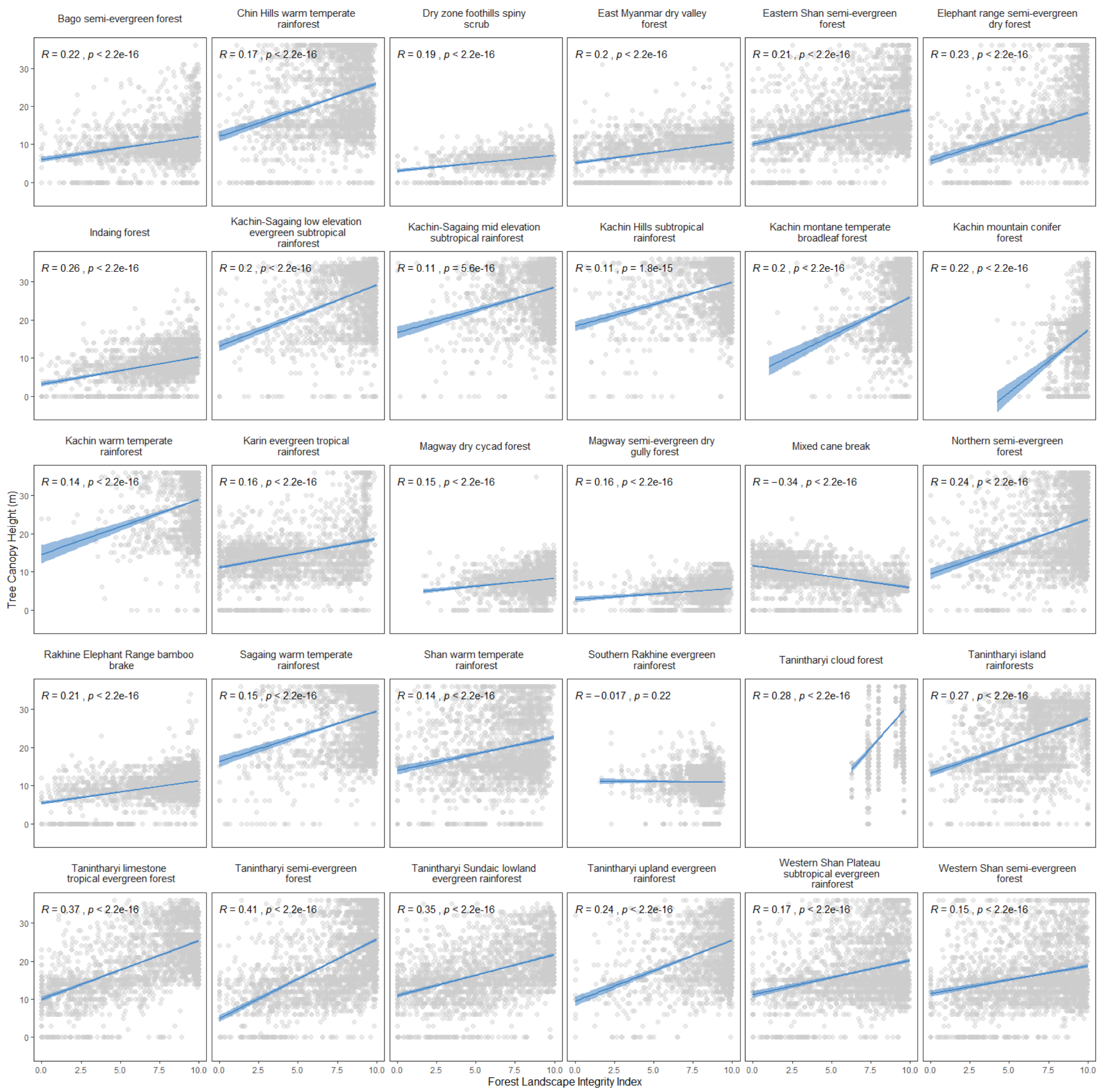

Supplementary Figure 7. Correlation between Forest Landscape Integrity Index and mean canopy height for forest ecosystems in Myanmar. N = 2500 independent samples for each forest ecosystem. Correlation was measured using a two-sided Kendall's T coefficient. Coloured lines are linear model fits with 95% confidence intervals (shaded regions).

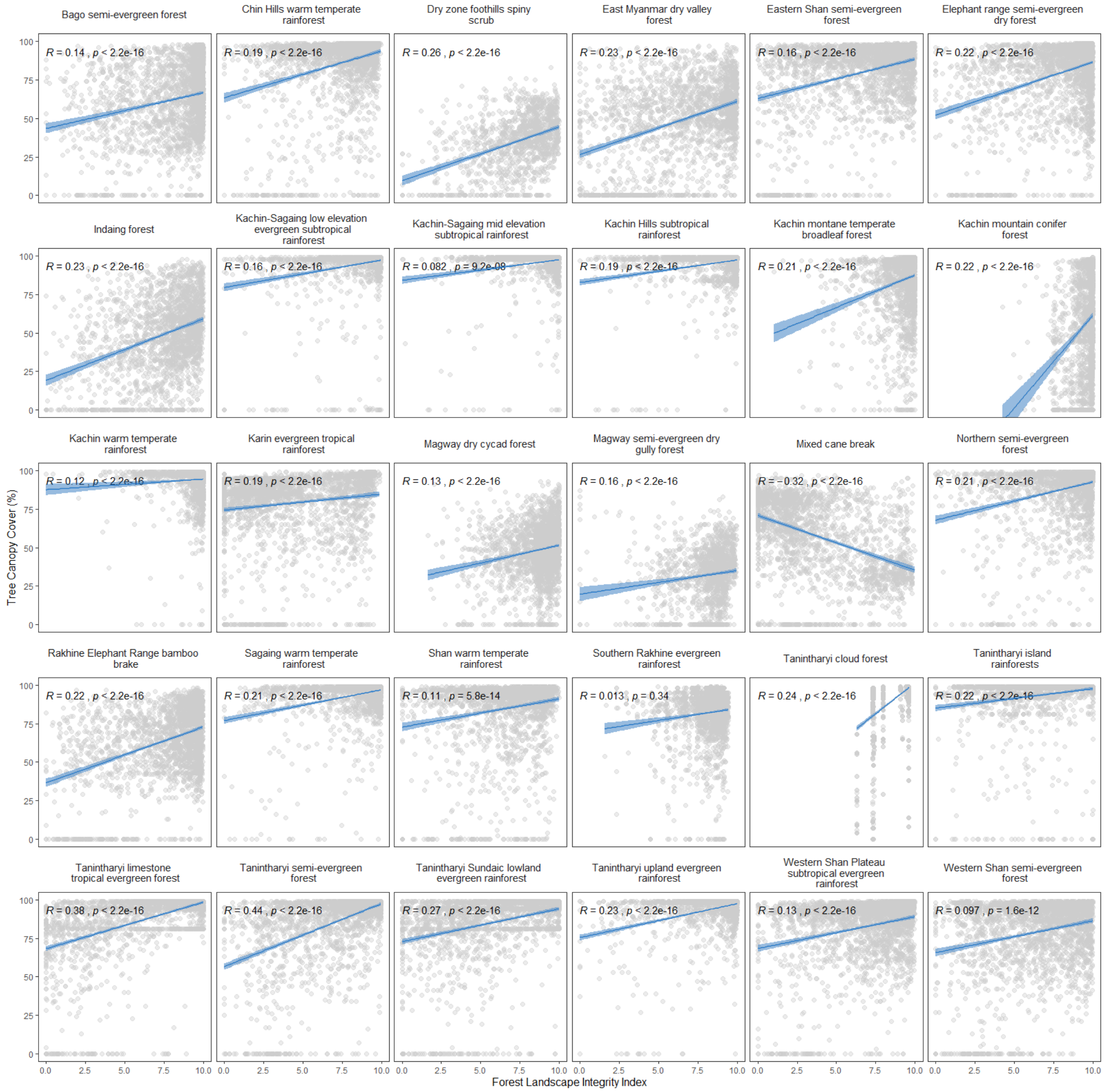

Supplementary Figure 8. Correlation between Forest Landscape Integrity Index and tree canopy cover for forest ecosystems in Myanmar. N = 2500 independent samples for each forest ecosystem. Correlation was measured using a two-sided Kendall's T coefficient. Coloured lines are linear model fits with 95% confidence intervals (shaded regions).

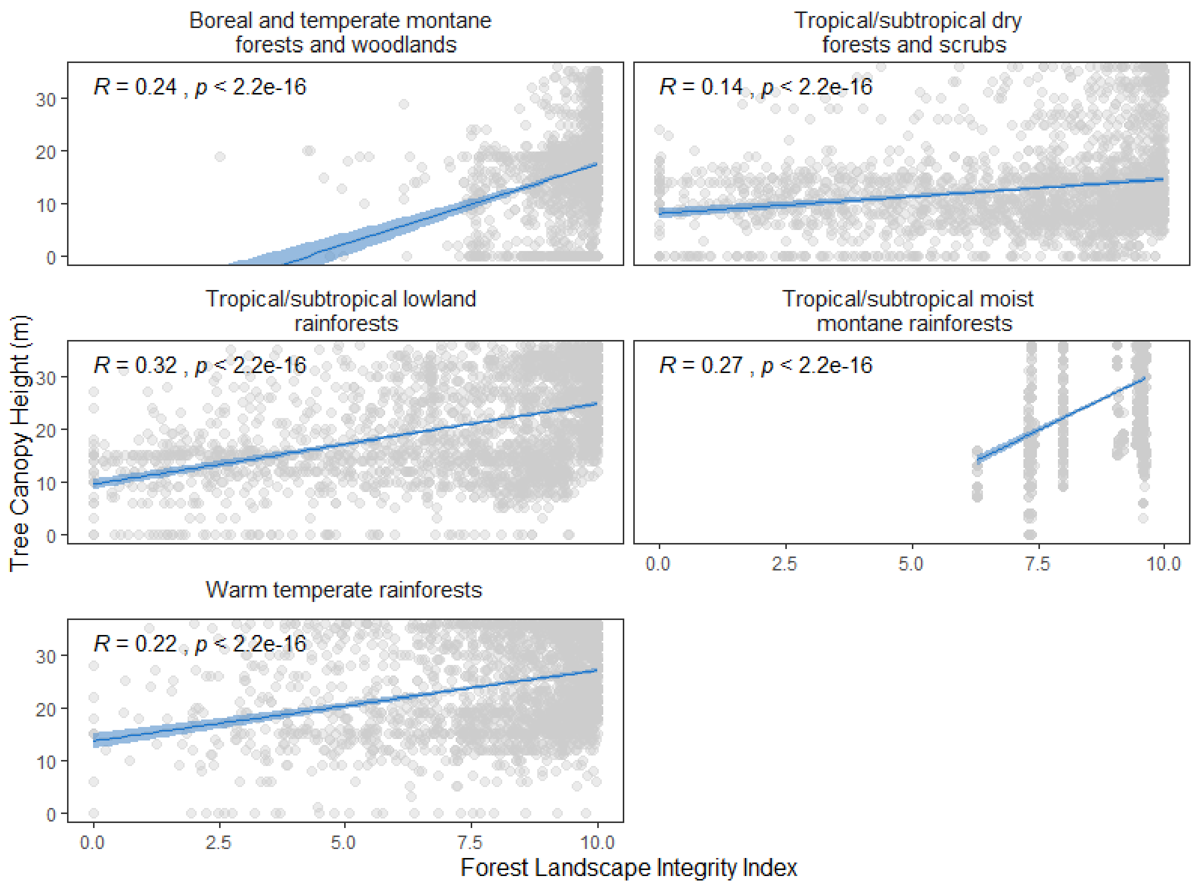

Supplementary Figure 9. Correlation between Forest Landscape Integrity Index and mean canopy height for forest functional groups in Myanmar. N = 2500 independent samples for each forest functional group. Correlation was measured using a two-sided Kendall's T coefficient. Coloured lines are linear model fits with 95% confidence intervals (shaded regions).

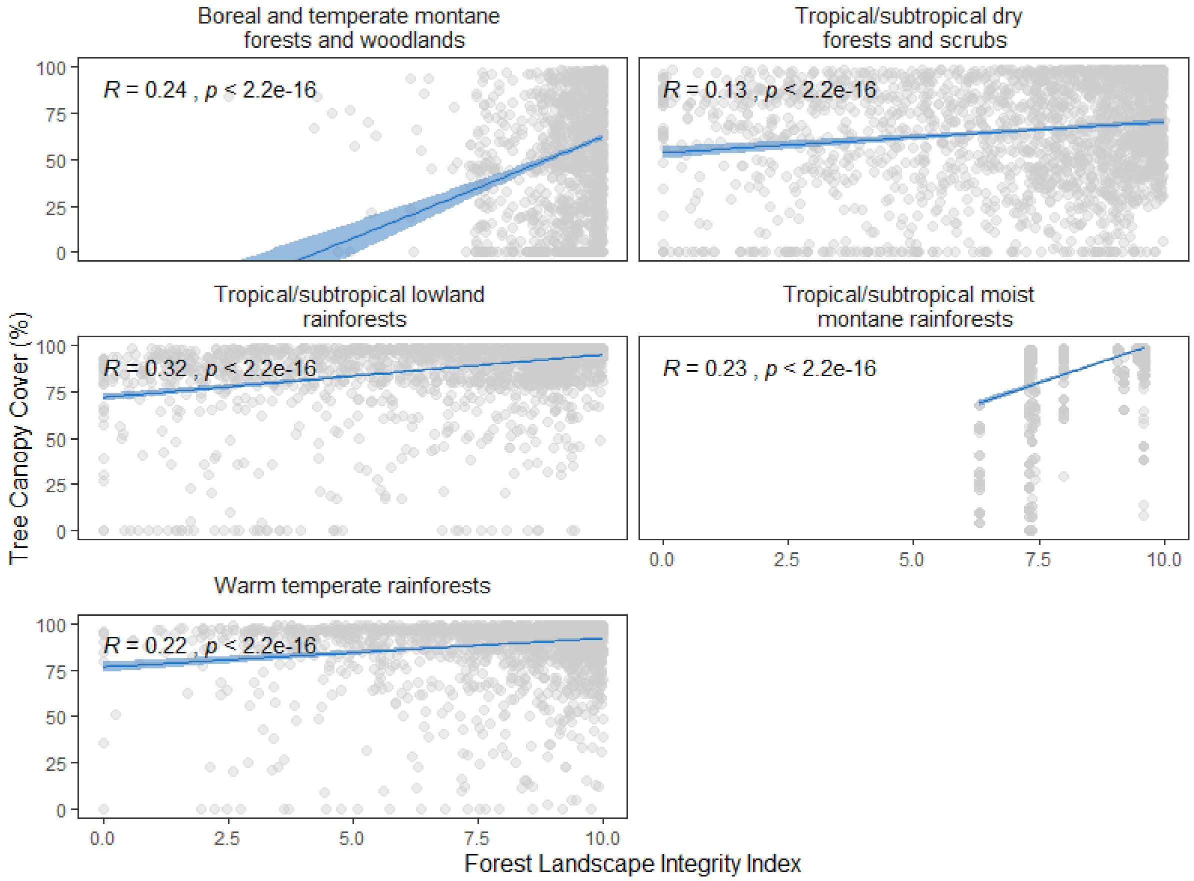

Supplementary Figure 10. Correlation between Forest Landscape Integrity Index and tree canopy cover for forest functional groups in Myanmar. N = 2500 independent samples for each forest functional group. Correlation was measured using a two-sided Kendall's T coefficient. Coloured lines are linear model fits with 95% confidence intervals (shaded regions).

Supplementary References

1 Hansen, M. C. *et al.* High-Resolution Global Maps of 21st-Century Forest Cover Change. *Science* **342**, 850-853, doi:10.1126/science.1244693 (2013).

2 Gibson, L. *et al.* Primary forests are irreplaceable for sustaining tropical biodiversity. *Nature* **478**, 378-381 (2011).

3 Curtis, P. G., Slay, C. M., Harris, N. L., Tyukavina, A. & Hansen, M. C. Classifying drivers of global forest loss. *Science* **361**, 1108-1111, doi:10.1126/science.aau3445 (2018).

4 Laestadius, L. *et al.* Opportunities for forest landscape restoration. *Unasylva* **62**, 238 (2011).

5 ESA. Land Cover CCI Product User Guide Version 2. Tech. Rep., (2017).

6 Theobald, D. M. A general model to quantify ecological integrity for landscape assessments and US application. *Landscape Ecol.* **28**, 1859-1874 (2013).

7 Sanderson, E. W. *et al.* The human footprint and the last of the wild. *Bioscience* **52**, 891-904 (2002).

8 Venter, O. *et al.* Sixteen years of change in the global terrestrial human footprint and implications for biodiversity conservation. *Nature Communications* **7**, 12558 (2016).

9 Kennedy, C. M., Oakleaf, J. R., Theobald, D. M., Baruch-Mordo, S. & Kiesecker, J. Managing the middle: A shift in conservation priorities based on the global human modification gradient. *Global Change Biol.* **25**, 811-826 (2019).

10 Thompson, I. D. *et al.* An Operational Framework for Defining and Monitoring Forest Degradation. *Ecology and Society* **18** (2013).

11 Contributors. (ed OpenStreetMap) (2015).

12 Teluguntla, P. *et al.* (ed NASA EOSDIS Land Processes DAAC) (2016).

13 Haddad, N. M. *et al.* Habitat fragmentation and its lasting impact on Earth's ecosystems. *Science Advances* **1**, e1500052, doi:10.1126/sciadv.1500052 (2015).

14 Laurance, W. F. *et al.* Ecosystem decay of Amazonian forest fragments: a 22-year investigation. *Conserv. Biol.* **16**, 605-618 (2002).

15 Zimmerman, B. L. & Kormos, C. F. Prospects for Sustainable Logging in Tropical Forests. *Bioscience* **62**, 479-487, doi:10.1525/bio.2012.62.5.9 (2012).

16 Cochrane, M. A. & Laurance, W. F. Fire as a large-scale edge effect in Amazonian forests. *Journal of Tropical Ecology* **18**, 311-325, doi:10.1017/S0266467402002237 (2002).

17 Peres, C. A., Emilio, T., Schietti, J., Desmoulière, S. J. M. & Levi, T. Dispersal limitation induces long-term biomass collapse in overhunted Amazonian forests. *Proceedings of the National Academy of Sciences* **113**, 892-897, doi:10.1073/pnas.1516525113 (2016).

18 Chaplin-Kramer, R. *et al.* Degradation in carbon stocks near tropical forest edges. *Nature Communications* **6**, 10158, doi:10.1038/ncomms10158 https://www.nature.com/articles/ncomms10158#supplementary-information (2015).

19 Tulloch, A. I., Gordon, A., Runge, C. A. & Rhodes, J. R. Integrating spatially realistic infrastructure impacts into conservation planning to inform strategic environmental assessment. *Conservation Letters* **12**, e12648 (2019).

20 Benítez-López, A., Alkemade, R. & Verweij, P. A. The impacts of roads and other infrastructure on mammal and bird populations: a meta-analysis. *Biol. Conserv.* **143**, 1307-1316 (2010).

21 Shapiro, A. C., Aguilar-Amuchastegui, N., Hostert, P. & Bastin, J.-F. Using fragmentation to assess degradation of forest edges in Democratic Republic of Congo. *Carbon Balance and Management* **11**, 11, doi:10.1186/s13021-016-0054-9 (2016).

22 Broadbent, E. N. *et al.* Forest fragmentation and edge effects from deforestation and selective logging in the Brazilian Amazon. *Biol. Conserv.* **141**, 1745-1757 (2008).
23 Maisels, F. *et al.* Devastating Decline of Forest Elephants in Central Africa. *PLOS ONE* **8**, e59469, doi:10.1371/journal.pone.0059469 (2013).
24 Benítez-López, A., Santini, L., Schipper, A. M., Busana, M. & Huijbregts, M. A. J. Intact but empty forests? Patterns of hunting-induced mammal defaunation in the tropics. *PLOS Biology* **17**, e3000247, doi:10.1371/journal.pbio.3000247 (2019).
25 Barrington-Leigh, C. & Millard-Ball, A. The world's user-generated road map is more than 80% complete. *PLOS ONE* **12**, e0180698, doi:10.1371/journal.pone.0180698 (2017).
26 Hughes, A. C. Have Indo-Malaysian forests reached the end of the road? *Biol. Conserv.* **223**, 129-137, doi:https://doi.org/10.1016/j.biocon.2018.04.029 (2018).
27 Tierney, G. L., Faber-Langendoen, D., Mitchell, B. R., Shriver, W. G. & Gibbs, J. P. Monitoring and evaluating the ecological integrity of forest ecosystems. *Frontiers in Ecology and the Environment* **7**, 308-316, doi:10.1890/070176 (2009).
28 Shapiro, A. *et al.* Forest condition in the Congo Basin for the assessment of ecosystem conservation status. *bioRxiv*, 2020.2003.2025.008110, doi:10.1101/2020.03.25.008110 (2020).
29 Murray, N., Keith, D.A., Tizard, R., Duncan, R., Htut, W., Hlaing, N, Oo, A.H., Ya, K.Z., Grantham, H. (ed Figshare) (2020).
30 Xu, L. *et al.* Spatial Distribution of Carbon Stored in Forests of the Democratic Republic of Congo. *Scientific Reports* **7**, 15030, doi:10.1038/s41598-017-15050-z (2017).
31 Potapov, P. V. *https://glad.umd.edu/dataset/mekong*.
32 Murray, N. J. *et al. Threatened ecosystems of Myanmar. An IUCN Red List of Ecosystems Assessment*. V1.0 edn, (2020).
33 Lamarche, C. *et al.* Compilation and validation of SAR and optical data products for a complete and global map of inland/ocean water tailored to the climate modeling community. *Remote Sensing* **9**, 36 (2017).
34 Gumma, M. *et al.* NASA Making Earth System Data Records for Use in Research Environments (MEaSUREs) Global Food Security-support Analysis Data (GFSAD) Cropland Extent 2015 South Asia, Afghanistan, Iran 30 m V001. (2017).
35 Massey, R. *et al.* (ed NASA EOSDIS Land Processes DAAC) (2017).
36 Oliphant, A. *et al.* NASA Making Earth System Data Records for Use in Research Environments (MEaSUREs) Global Food Security-support Analysis Data (GFSAD) Cropland Extent 2015 Southeast Asia 30 m V001. (2017).
37 Phalke, A. *et al.* (ed NASA EOSDIS Land Processes DAAC) (2017).
38 Teluguntla, P. *et al.* NASA Making Earth System Data Records for Use in Research Environments (MEaSUREs) Global Food Security-support Analysis Data (GFSAD) Cropland Extent 2015 Australia, New Zealand, China, Mongolia 30 m V001. (2017).
39 Xiong, J. *et al.* NASA Making Earth System Data Records for Use in Research Environments (MEaSUREs) Global Food Security-support Analysis Data (GFSAD) Cropland Extent 2015 Africa 30 m V001. (2017).
40 Zhong, Y. *et al.* (ed NASA EOSDIS Land Processes DAAC) (2017).
41 Pekel, J.-F., Cottam, A., Gorelick, N. & Belward, A. S. High-resolution mapping of global surface water and its long-term changes. *Nature* **540**, 418-422 (2016).
