# Supplementary material for: Anthropogenic modification of forests means only 40% of remaining forests have high ecosystem integrity
Source: Nat Commun. 2020 Dec 8;11:5978. doi: 10.1038/s41467-020-19493-3 (PMC7723057; doi:10.1038/s41467-020-19493-3)
Supplement: Supplementary file 2 — Reporting Summary [file 41467_2020_19493_MOESM2_ESM.pdf]

nature research

Corresponding author(s): *Double-blind peer review submissions: write DBPR and your manuscript number here instead of author names.*

Last updated by author(s): *YYYY-MM-DD*

Reporting Summary

Nature Research wishes to improve the reproducibility of the work that we publish. This form provides structure for consistency and transparency in reporting. For further information on Nature Research policies, see our Editorial Policies and the Editorial Policy Checklist.

Statistics

For all statistical analyses, confirm that the following items are present in the figure legend, table legend, main text, or Methods section.

n/a	Confirmed	
☐	☒	The exact sample size (n) for each experimental group/condition, given as a discrete number and unit of measurement
☒	☐	A statement on whether measurements were taken from distinct samples or whether the same sample was measured repeatedly
☒	☐	The statistical test(s) used AND whether they are one- or two-sided<br>*Only common tests should be described solely by name; describe more complex techniques in the Methods section.*
☒	☐	A description of all covariates tested
☒	☐	A description of any assumptions or corrections, such as tests of normality and adjustment for multiple comparisons
☐	☒	A full description of the statistical parameters including central tendency (e.g. means) or other basic estimates (e.g. regression coefficient) AND variation (e.g. standard deviation) or associated estimates of uncertainty (e.g. confidence intervals)
☐	☒	For null hypothesis testing, the test statistic (e.g. F, t, r) with confidence intervals, effect sizes, degrees of freedom and P value noted<br>*Give P values as exact values whenever suitable.*
☒	☐	For Bayesian analysis, information on the choice of priors and Markov chain Monte Carlo settings
☒	☐	For hierarchical and complex designs, identification of the appropriate level for tests and full reporting of outcomes
☒	☐	Estimates of effect sizes (e.g. Cohen's d, Pearson's r), indicating how they were calculated

Our web collection on statistics for biologists contains articles on many of the points above.

Software and code

Policy information about availability of computer code

Data collection	No software was used for data collection
Data analysis	The analysis was completed in Google Earth Engine. There are no versions of this software but it was run on 07/21/2019. The code for Google Earth Engine is available upon any reasonable request. ESRI Arcmap was used to compute the spatial summaries.

For manuscripts utilizing custom algorithms or software that are central to the research but not yet described in published literature, software must be made available to editors and reviewers. We strongly encourage code deposition in a community repository (e.g. GitHub). See the Nature Research guidelines for submitting code & software for further information.

Data

Policy information about availability of data

All manuscripts must include a data availability statement. This statement should provide the following information, where applicable:

- Accession codes, unique identifiers, or web links for publicly available datasets
- A list of figures that have associated raw data
- A description of any restrictions on data availability

Data availability statement: The authors declare that all data supporting the findings of this study are available at www.forestlandscapeintegrity.com. The datasets used to develop the Forest Landscape Integrity Index can be found at the following websites: tree cover and loss http://earthenginepartners.appspot.com/science-2013-global-forest, tree cover loss driver https://data.globalforestwatch.org/datasets/f2b7de1bdde04f7a9034ecb363d71f0e, potential forest cover https://data.globalforestwatch.org/datasets/potential-forest-coverage ESA-CCI Land Cover https://maps.elie.ucl.ac.be/CCI/viewer/index.php Open Street Maps https://www.openstreetmap.org, croplands https://lpdaac.usgs.gov/news/release-of-gfsad-30-meter-cropland-extent-products/, surface water https://global-surface-water.appspot.com/, protected areas https://www.protectedplanet.net/en.

Field-specific reporting

Please select the one below that is the best fit for your research. If you are not sure, read the appropriate sections before making your selection.

☐ Life sciences ☐ Behavioural & social sciences ☒ Ecological, evolutionary & environmental sciences

For a reference copy of the document with all sections, see nature.com/documents/nr-reporting-summary-flat.pdf

Ecological, evolutionary & environmental sciences study design

All studies must disclose on these points even when the disclosure is negative.

Study description	The study developed a composite index that combines numerous datasets to measure the integrity of the worlds forests.
Research sample	The Forest Landscape Integrity Index was based on the following datasets: tree cover, tree cover loss, driver of tree cover loss, infrastructure, cropland, cropland intensity, and water surface data. These are all publically available datasets and their references can be found within Supplementary Table 1 and the data availability statement.
Sampling strategy	These datasets were chosen as they were known in the literature to be key drivers of forest integrity. Other datasets on human population density, night time lights were also considered but were found to be highly correlated and therefore confounded with other variables in the index
Data collection	Data was collecting from various open sources datasets and stored within google earth engine by Adam Duncan
Timing and spatial scale	The index represents the state of the forests for the start of 2019. Most of the datasets record the state of various factors at some time during 2018. The spatial scale is all of the worlds forests at a 300m resolution.
Data exclusions	There were no data exclusions.
Reproducibility	The datasets are uploaded onto the Google Earth Engine library. We can share the code that is then used to do the calculations on request. But given it is a static code there is an identical result each time the code is run.
Randomization	There was no randomization as this was a spatial analysis.
Blinding	Blinding was not possible given the nature of this spatial analysis.

Did the study involve field work? ☐ Yes ☒ No

Reporting for specific materials, systems and methods

We require information from authors about some types of materials, experimental systems and methods used in many studies. Here, indicate whether each material, system or method listed is relevant to your study. If you are not sure if a list item applies to your research, read the appropriate section before selecting a response.

Materials & experimental systems

n/a	Involved in the study	
☒	☐	Antibodies
☒	☐	Eukaryotic cell lines
☒	☐	Palaeontology and archaeology
☒	☐	Animals and other organisms
☒	☐	Human research participants
☒	☐	Clinical data
☒	☐	Dual use research of concern

Methods

n/a	Involved in the study	
☒	☐	ChIP-seq
☒	☐	Flow cytometry
☒	☐	MRI-based neuroimaging
